# Immune response caused by M1 macrophages elicits atrial fibrillation-like phenotypes in coculture model with isogenic hiPSC-derived cardiomyocytes

**DOI:** 10.1186/s13287-024-03814-0

**Published:** 2024-09-04

**Authors:** Thomas Hutschalik, Ozan Özgül, Marilù Casini, Brigitta Szabó, Rémi Peyronnet, Óscar Bártulos, Mariana Argenziano, Ulrich Schotten, Elena Matsa

**Affiliations:** 1 Ncardia Services B.V, J.H. Oortweg 21, 2333 CH Leiden, The Netherlands; 2 Dept. of Physiology, Cardiovascular Research Institute Maastricht, Maastricht, The Netherlands; 3 Regenerative Medicine and Heart Transplantation Unit, Instituto de Investigación Sanitaria La Fe, 46026 Valencia, Spain; 4 Institute for Experimental Cardiovascular Medicine, University Heart Center Freiburg Bad Krozingen and Faculty of Medicine, Freiburg im Breisgau, 79110 Germany; 5 Dept. of Cardiology, Maastricht University Medical Center, Maastricht, The Netherlands; 6 Rue Edouard Belin 2, 1435 CellisticMont-Saint-Guibert, Belgium; 7 School of Biochemistry and Cell Biology, University College Cork, Cork, Ireland; 8 National Institute for Bioprocessing Research and Training, Dublin, Ireland

**Keywords:** Atrial fibrillation, Atrial cardiomyocytes, Macrophages, Inflammation, hiPSC, Disease modeling

## Abstract

**Background:**

Atrial fibrillation has an estimated prevalence of 1.5–2%, making it the most common cardiac arrhythmia. The processes that cause and sustain the disease are still not completely understood. An association between atrial fibrillation and systemic, as well as local, inflammatory processes has been reported. However, the exact mechanisms underlying this association have not been established. While it is understood that inflammatory macrophages can influence cardiac electrophysiology, a direct, causative relationship to atrial fibrillation has not been described. This study investigated the pro-arrhythmic effects of activated M1 macrophages on human induced pluripotent stem cell (hiPSC)-derived atrial cardiomyocytes, to propose a mechanistic link between inflammation and atrial fibrillation.

**Methods:**

Two hiPSC lines from healthy individuals were differentiated to atrial cardiomyocytes and M1 macrophages and integrated in an isogenic, pacing-free, atrial fibrillation-like coculture model. Electrophysiology characteristics of cocultures were analysed for beat rate irregularity, electrogram amplitude and conduction velocity using multi electrode arrays. Cocultures were additionally treated using glucocorticoids to suppress M1 inflammation. Bulk RNA sequencing was performed on coculture-isolated atrial cardiomyocytes and compared to meta-analyses of atrial fibrillation patient transcriptomes.

**Results:**

Multi electrode array recordings revealed M1 to cause irregular beating and reduced electrogram amplitude. Conduction analysis further showed significantly lowered conduction homogeneity in M1 cocultures. Transcriptome sequencing revealed reduced expression of key cardiac genes such as *SCN5A*, *KCNA5*, *ATP1A1, and GJA5* in the atrial cardiomyocytes*.* Meta-analysis of atrial fibrillation patient transcriptomes showed high correlation to the in vitro model. Treatment of the coculture with glucocorticoids showed reversal of phenotypes, including reduced beat irregularity, improved conduction, and reversed RNA expression profiles.

**Conclusions:**

This study establishes a causal relationship between M1 activation and the development of subsequent atrial arrhythmia, documented as irregularity in spontaneous electrical activation in atrial cardiomyocytes cocultured with activated macrophages. Further, beat rate irregularity could be alleviated using glucocorticoids. Overall, these results point at macrophage-mediated inflammation as a potential AF induction mechanism and offer new targets for therapeutic development. The findings strongly support the relevance of the proposed hiPSC-derived coculture model and present it as a first of its kind disease model.

**Supplementary Information:**

The online version contains supplementary material available at 10.1186/s13287-024-03814-0.

## Background

Atrial fibrillation (AF) has an estimated prevalence of 1.5–2%, a number expected to double in coming decades[1]*.* Current treatment options, such as anti-arrhythmic drugs, cardioversion, and ablation show limited efficacy, requiring repeat interventions in up to 45% of cases[2, 3]. This necessitates better mechanistic understanding of arrhythmia occurrence to improve treatments.

The mechanisms that cause and sustain AF are still not completely understood but several studies point to structural or electrophysiological abnormalities of the atria, possibly linked to inflammation[1, 4]. Such studies have demonstrated significant increases in inflammation marker serum levels and number of pro-inflammatory macrophages in AF patient atrial biopsies and animal models[5, 6]. While inflammation has been strongly associated with cardiac arrhythmia, consensus is lacking on whether it is a cause or consequence[7, 8].

Lately, macrophages have garnered interest regarding their impact on cardiac electrophysiology[9]. Resident cardiac tissue macrophages make up 5–10% of all cells in the healthy heart, while cardiomyocytes constitute ~30% [10–13], and were shown to have functionalities beyond established roles in host defense. These include involvement in cardiac conduction by influencing pacemaker cells through gap junctions[14].

Specific links of macrophages to AF have so far only been shown in canine and mouse models where AF-like phenotypes, such as decreased atrial effective refractory period and L-type calcium currents (I _Ca-L_) were induced by burst pacing that in turn activated tissue resident macrophages[5, 15]. While human models of AF have been described using iPSC-derived cells[16], these have not addressed inflammatory disease causes. To date, no in vitro or in vivo model has presented macrophages as direct instigators of beat irregularity in atrial cells.

This study sought to investigate whether activated M1 macrophage-mediated inflammation can be a cause for AF-like cellular phenotypes, using an isogenic coculture model of atrial cardiomyocytes and macrophages derived from hiPSC. In electrophysiological measurements, macrophage activation led to beat rate irregularity and other electrophysiological perturbations, pointing at inflammation as a direct cause of arrhythmogenesis. Transcriptome analysis showed significant dysregulation in ion channels genes, including *SCN5A*, *KCNA5*, *ATP1A1,* and in *GJA5*, transcribing the atrial-specific gap junction Cx40. Transcriptional changes were significantly correlated to patient tissue data from AF clinical trials[17], demonstrating the physiological relevance of the in vitro hiPSC model. Moreover, anti-inflammatory compound intervention significantly alleviated beat irregularities, aligning with previous clinical findings[7, 18–20]. Anti-inflammatory agents further restored ion channel expression, confirming the direct impact of macrophage-induced inflammation on cardiomyocyte function.

## Results

### hiPSC-derived atrial-like cardiomyocytes and cardiac tissue resident macrophages form integrated coculture

Atrial-like cardiomyocytes and M1 macrophages were derived from two hiPSC lines and expressed high levels of lineage-specific markers (Figure S1, S2, and S3A,B). aCM also displayed action potential morphology characteristic of aCM in sharp electrode recording analysis (Figure S2B,C).

To define coculture conditions, it was important that both cell types maintained their identity and functionality in a common media formulation. The effect of supplemented cardiomyocyte medium on monocyte/macrophages monocultures was thus tested. The medium did not affect expression of cell identity markers CD14 (Figure S3C,D), Vimentin and CX3CR1 (Figure S3D,E) compared to monocyte medium. Further, activation of M0 monocytes to M1 macrophages was not adversely affected by supplemented cardiomyocyte medium (Figure S3D,E), nor was the transcription of M2-specific IL10 compared to monocyte Medium (Figure S3F)*.* CD68, a macrophage activation marker, was expressed in M1 while remaining absent in monocytes (Figure S3G). Activated M1 performed phagocytosis through phagosomes (Figure S4A). Congruently, cardiomyocyte medium allowed M1 cytokine transcription and release at comparable levels to monocyte medium (Figure S4B-E), absent in non-activated conditions.

**Fig. 1:**
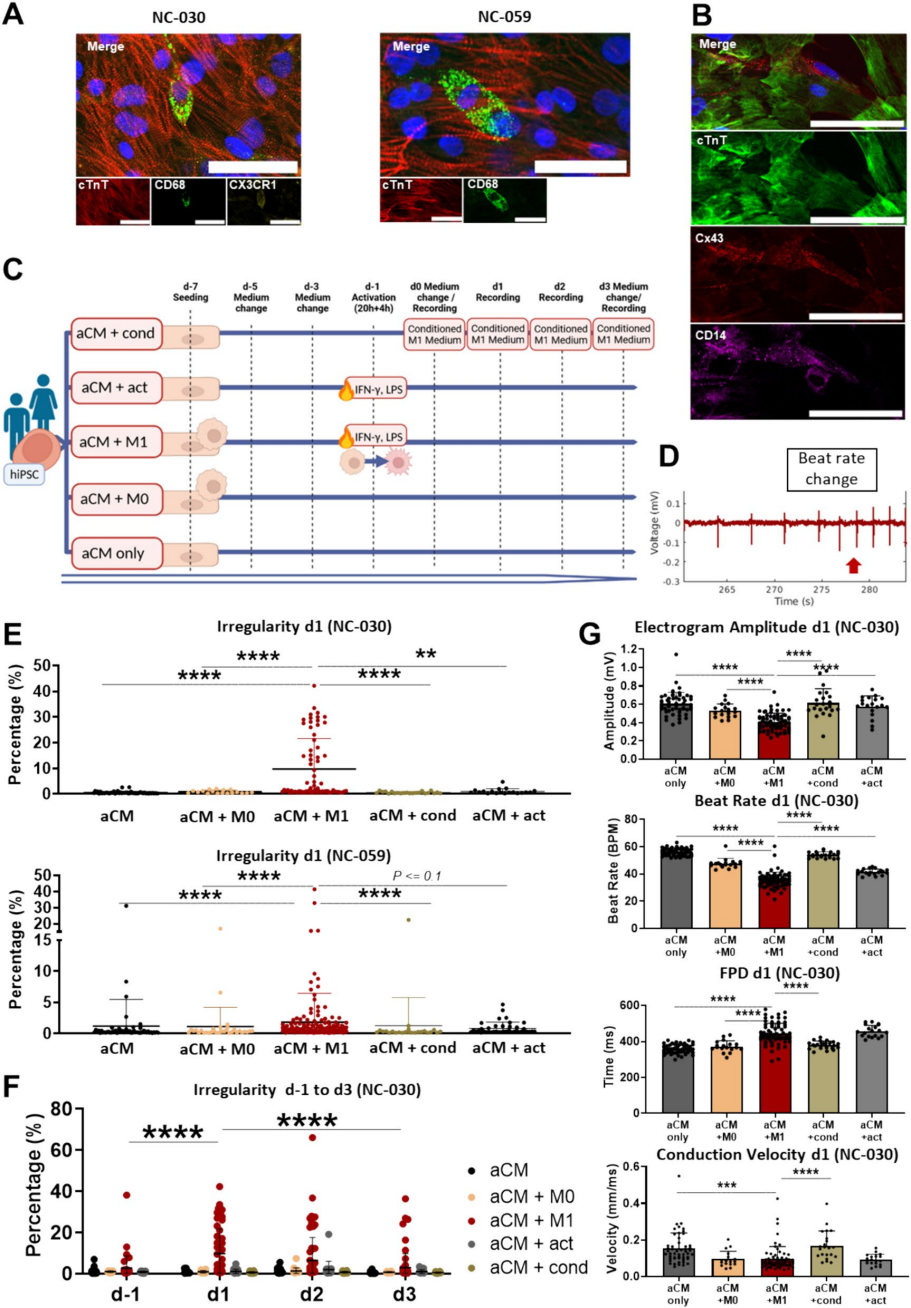
aCM + M1 coculture resulted in higher occurrence of arrhythmias and electrophysiological changes. **A** Immunofluorescence (IF) image of NC-030 coculture of aCM and M1 (d10) stained for cTnT, CX3CR1, CD68 and DAPI, showing M1 connected to aCM and expressing activation and tissue-resident markers; NC-059 coculture stained for cTnT, CD68 and DAPI (scale bars 50µm). **B** IF images of NC-030 aCM and M1 cocultures (d8) stained for cTnT, Cx43, CD14 and DAPI show CD14^+^ macrophage expressing Cx43 while in contact with aCM (scale bar 50µm). **C** Schematic of experimental schedule and conditions tested in MEA assay. Conditions were: atrial cardiomyocytes only (aCM only), aCM + M0 macrophages (aCM + M0), aCM + M1 macrophages (aCM + M1), aCM + M1 conditioned medium (aCM + cond) and aCM only with activation factors added (aCM + act) **D** Exemplary MEA trace, showing electrogram (i.e., sodium spikes) over time with sudden beat rate change in a NC-030 aCM + M1 sample. **E** Scatter dot plots showing beat irregularity on d1 after activation. Mann Whitney test used to compare conditions. **F** Scatter dot plot of NC-030 one day before activation (d-1) and up to 3 days thereafter (d1-3), showing highly significant increase of irregularity in aCM + M1 after activation and loss of irregularity over time. **G** Bar graphs of NC-030 on d1 after activation comparing electrogram amplitude, beat rate, FPD and conduction velocity between conditions

Using the supplemented cardiomyocyte medium, isogenic aCM and M1 formed functional cocultures (Video S1, Figure S4F). Cocultured macrophages showed characteristic spindle-like morphology and integrated within aCM monolayers[13, 14], establishing an in vitro coculture of pro-inflammatory M1 and aCM. Macrophages persisted in coculture with aCM and were able to undergo activation as described above, expressing activation marker CD68, cardiac tissue-resident macrophage marker CX3CR1 and macrophage marker CD14 (Fig. 1A, Figure S4G). Further, macrophages expressed gap junctions (Cx43)[14] adjacent to aCMs (Fig. 1B). In summary, a functional coculture model of aCM and M1 was developed, showing M1 integrating into aCM layers, while maintaining their subtype identity and functionality.

### Activated M1 induce electrophysiological abnormalities in aCM

To investigate the effects of M1 activation on aCM electrophysiology, five (co)culture conditions were studied. These included direct coculture of aCM and M1 (aCM + M1), and aCM cultured in M1 conditioned medium (aCM + cond), to investigate the effect of macrophage cytokine secretion without cell contact. Cytokine presence in supernatant was confirmed for IL-6 at d1 after activation (NC-030: 138 pg/ml ± 62, NC-059: 235 pg/ml ± 24, Figure S4C). Further, three controls were included: aCM only, to establish baseline conditions of aCM, aCM with added activation factors LPS and IFN-γ (aCM + act), to exclude effects of activation agents on aCM electrophysiology, and aCM + M0, to exclude the effect of non-inflammatory cells on electrophysiology (Fig. 1C). In cell lines tested (NC-030 and NC-059), cocultures of aCM + M1 resulted in arrhythmia-like changes (Fig. 1D), detected as significant beat rate irregularity during recordings compared to controls (mean beat irregularity NC-030: 9.7% ± 11.9 aCM + M1 vs. 0.9% ± 0.6 aCM + M0) (Fig. 1E).

Beat rate irregularity emerged after activation (d1) gradually decreasing over time. Cocultures were stable, i.e., not irregular, before activation (d-1). This suggested that beat rate irregularity was connected to activation of macrophages in direct contact with aCM (Fig. 1F). Beat irregularity was supported as a surrogate measurement of pro-arrhythmia, by showing a dose-dependent increase of beat irregularity after treating aCM with the known pro-arrhythmic compound, ivabradine (Figure S4H). Arrhythmia and beat irregularity induction through tachycardia was further investigated through isoproterenol and aconitine addition. Isoproterenol addition resulted in a dose-dependent increase in beat rate (Figure S4I), with 1 µM isoproterenol causing a significant increase in beat irregularity (66%±23) (Figure S4J). Treatment using aconitine resulted in tachycardic (~200BPM) aCM at 5 µM (Figure S4K), which congruently presented emerging arrhythmias (Figure S4L).

M1 persisted in coculture throughout recordings, and retained their activated, tissue-resident subtype as confirmed by immunofluorescence (IF) (Figure S4M). The emergence of irregularity depended on macrophages being seeded simultaneously with aCM, while sequential addition of the same number of macrophages to the entire well resulted in no arrhythmia (Figure S4N). Simultaneous seeding of M1 and aCM confined cells to the same area on the electrodes leading to closely integrated layers. Sequentially added macrophages attached to areas not covered by aCM, resulting in a reduced number of M1 connecting to aCM. This further pointed towards direct cell contact leading to emerging phenotypes and not only cytokine secretion. Interestingly, doubling the number of macrophages during simultaneous seeding did not significantly increase irregularity, proposing a non-linear relation of M1 activation and subsequent effects. This suggested beat irregularity as being non-dependent on disrupting the physical interaction between aCM (Figure S4N).

Electrogram amplitude was also significantly lower in aCM + M1 (0.41 mV ± 0.09) compared to other conditions (aCM + M0 0.53 mV ± 0.08; all NC-030 mean) (Fig. 1G, Figure S4O), indicating a reduced depolarization potential in aCM + M1 cocultures. Electrogram amplitude was identified primarily as sodium current (*I*_Na_) by amplitude reduction following addition of the *I*_Na_ antagonist, flecainide (Figure S4P).

Further electrophysiological parameters showed changes not confined to M1 activation. For example, on d1, conduction velocity was significantly lower in aCM + M1 (0.1 mm/ms ± 0.07), aCM + M0 (0.1 mm/ms ± 0.04), and aCM + act (0.09 mm/ms ± 0.03), compared to aCM only (0.15 mm/m ± 0.09 s) and aCM + cond (0.17 mm/ms ± 0.08) (Fig. 1G, Figure S4O). Reduced conduction velocity in aCM + M1 was congruent with reduced electrogram amplitude. Beat rate was lower in activated conditions (aCM + M1 (35.6 BPM ± 5.7), aCM + act (41.5 BPM ± 2.2)), while conditioned medium showed no beat rate reduction (54 BPM ± 2.2; all NC-030 mean) (Fig. 1G, Figure S4O).

**Fig. 2:**
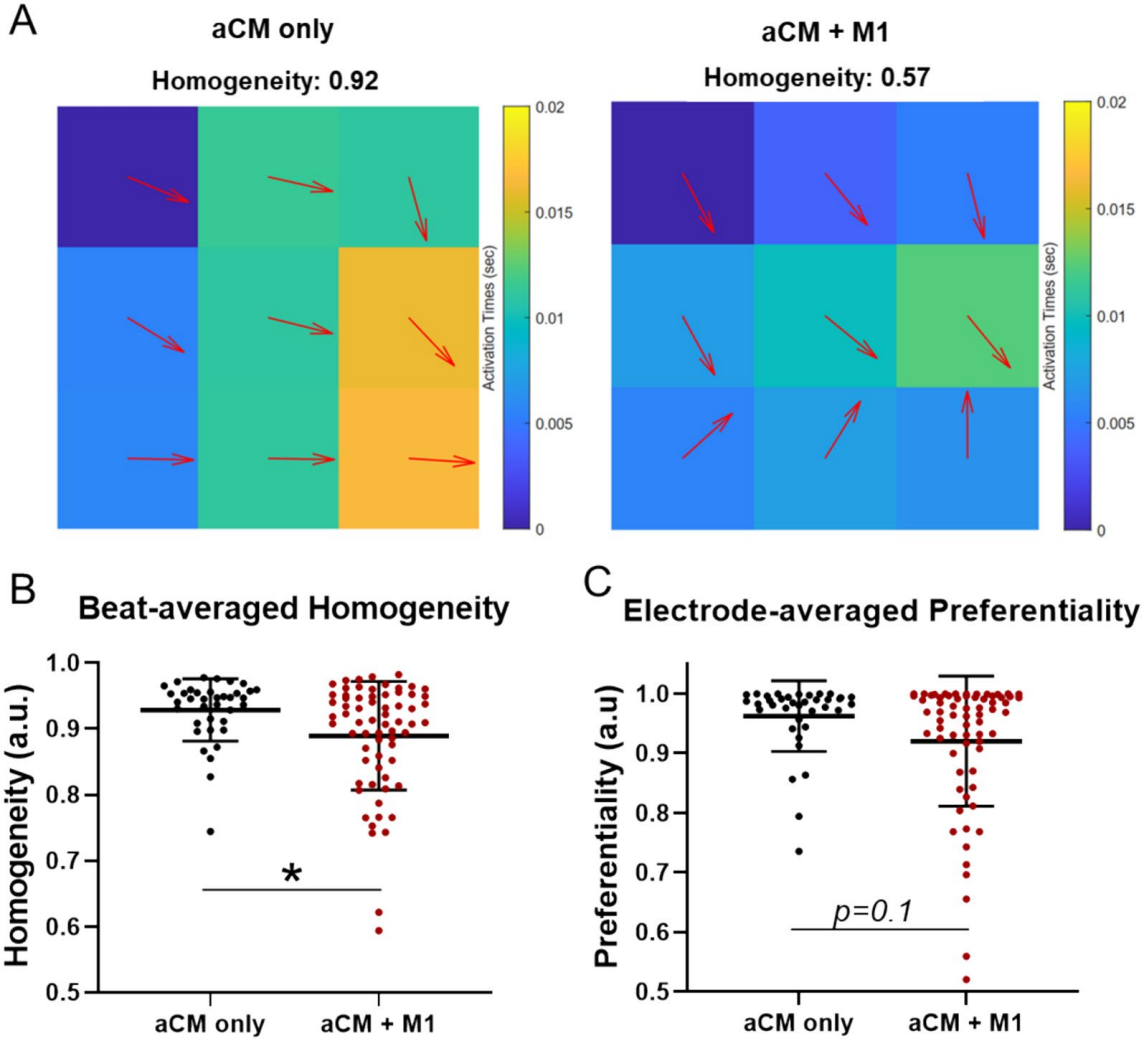
aCM and M1 cocultures altered electrical conduction, resulting in lower conduction homogeneity. **A** Representative activation time maps of a single beat for aCM only and aCM + M1 of a MEA recording. Colors denote the timepoint of activation for each electrode and vectors (arrows) represent the direction of conduction of each electrode. Homogeneity signifies the similarity of arrow (conduction) angular direction, 1 being all conduction traveling along the same angle. aCM + M1 shows lower homogeneity (0.57 a.u.) than aCM only (0.92 a.u.) in this representative mapping, indicating non-uniform conduction. **B** Scatter plot of beat-averaged homogeneity for aCM only and aCM + M1, each dot representing a whole recording per sample with homogeneity for each beat being averaged across the recording. (Mann–Whitney test). **C** Scatter plot of electrode-average preferentiality for aCM only and aCM + M1. Preferentiality represents the change of vector direction (conduction direction) of a single electrode over time. A value of 1 represents the direction of conduction being unchanged over time. Each dot in the graph represents a whole recording of per sample, with the preferentiality of all electrodes from one sample averaged. (Mann–Whitney test)

Conduction analysis based on MEA recordings was used to investigate beat-averaged homogeneity (i.e., uniformity of conduction direction between all electrodes, averaged for each beat) of aCM only and aCM + M1 conditions (Fig. 2A). Consistent with beat irregularity, homogeneity was significantly lower in the aCM + M1 coculture condition compared to aCM only (0.89 a.u., 0.93 a.u. respectively) (Fig. 2B). These emerging conduction disturbances, also observed in AF, are likely linked to reduced depolarization and slowed conduction. Electrode-averaged preferentiality (i.e., the consistency of conduction direction of each electrode, averaged for each electrode) showed no significant reduction for aCM + M1, but a lower average was observed compared to the control (0.92 a.u., 0.96 a.u. respectively), (Fig. 2C).

In conclusion, aCM coculture with M1 led to electrophysiological abnormalities, shown by an increase in beat irregularity, reduction of electrogram amplitude and increase in conduction heterogeneity. This effect was absent in M1 supernatant-treated aCM (aCM + cond) and other control conditions.

### Direct coculture with M1 causes differential expression of inflammation-related genes in aCM; resembling clinical, paroxysmal AF human tissue profiles

**Fig. 3:**
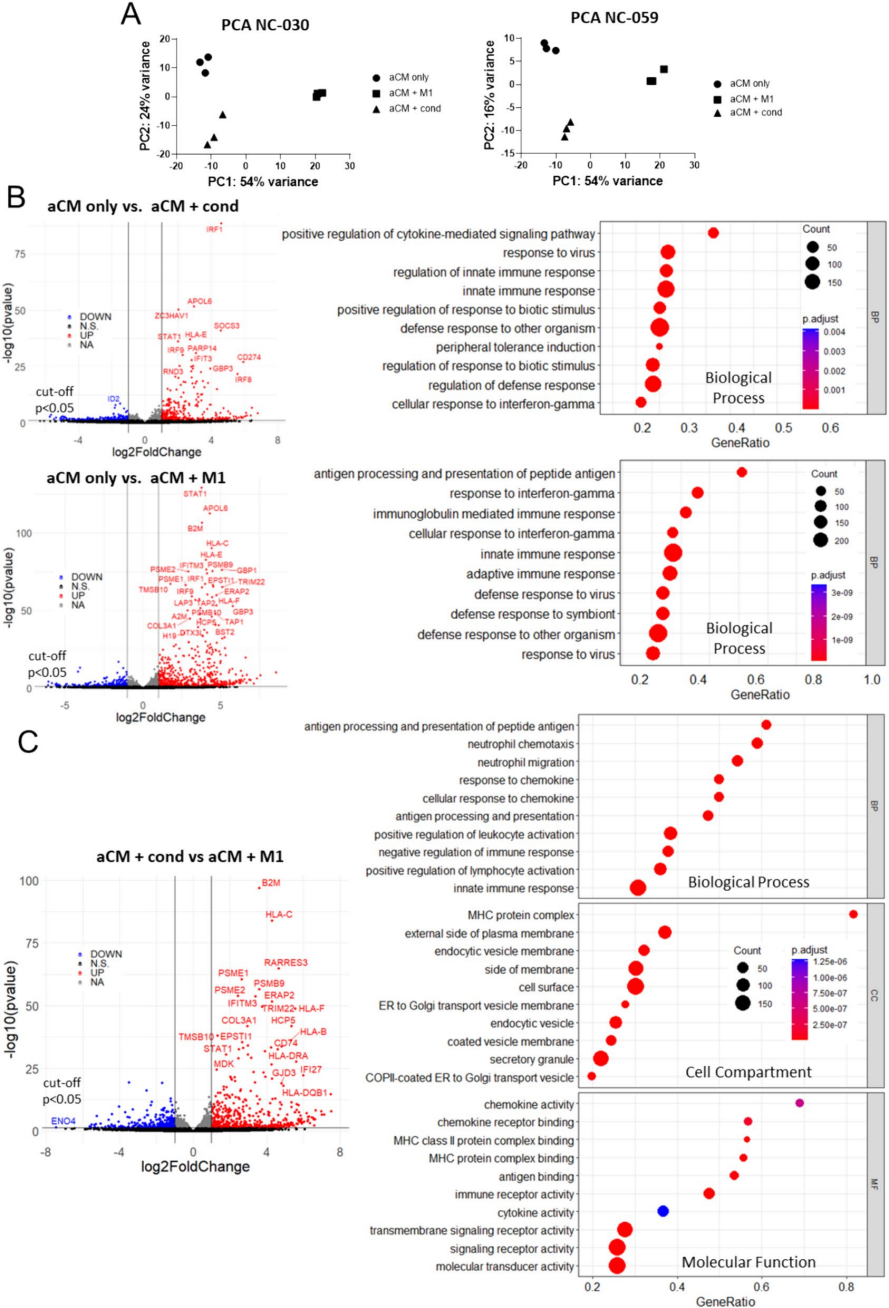
RNA-seq reveals increased inflammatory gene expression in aCM + M1 coculture compared to M1 conditioned medium-treated aCM. **A** PCA plots of RNA-seq data for NC-030 and NC-059 showing aCM only, aCM + M1 and aCM + cond separated according to principal components. **B** RNA sequencing data for NC-059 showing volcano and dot plots comparing aCM only vs. aCM + cond and aCM only vs. aCM + M1. Volcano plots show genes significantly (p < 0.05) changed as up (fold change > 1, red) or down regulated (fold change < -1, blue). Gene ontology analysis shows the most differentially impacted biological processes between conditions, primarily including inflammation-related processes. **C** RNA-seq data for NC-059 showing volcano and dot plots for aCM + cond vs. aCM + M1. Dot plots showing biological processes (BP), cell compartments (CC) and molecular functions (MF)

Principal component analysis (PCA) based on RNA-seq showed aCM only, aCM + cond and aCM + M1 clustering into distinct groupings, with nearly identical principal component variance distributions for both lines tested. Notably, principal component 1 (PC1) separated aCM + M1 from the other two conditions (PC1 variance NC-030: 54%, NC-059 54%), showing the direct coculture to have a stronger influence on variance than the aCM + cond condition (Fig. 3A). This indicated distinct effects on aCM caused by M1 coculture and M1 supernatant treatment. Low or undetectable expression levels of macrophage markers (e.g., CD14, CD86) showed successful depletion of macrophages from the cocultures prior to RNA-seq (Figure S5A). Direct comparison between aCM only and aCM + cond revealed prominent upregulation of genes (genes upregulated NC-030: 1174; NC-059: 677), that were nevertheless not sufficient to cause apparent electrophysiological remodelling. Most distinctly affected were inflammation-related genes, including Interferon Regulatory Factor 1 (*IRF-1*; 4.8 (NC-030), 4.6 (NC-059) log_2_-FoldChange), a transcriptional activator which stimulates immune response, including transcription of IFN-inducible genes[21]*.*

aCM + M1 condition showed 1,486 significantly upregulated genes for NC-030 and 1,241 for NC-059 compared to aCM only, including foremost inflammation-related genes e.g., *A2M* and *HLA*-C. Alpha-2-Macroglobulin (*A2M*; 2.8 (NC-030) 2.9 (NC-059) log_2_-FoldChange) is an inhibitor and transporter of inflammatory cytokines, able to disrupt inflammatory cascades[22]. Major Histocompatibility Complex, Class I, C (*HLA-C*; 4.7 (NC-030), 4.4 (NC-059) log_2_-FoldChange) is part of the MHC group, tasked with presenting pathogen fragments to immune cells. Gene Ontology (GO) analysis showed inflammation-related biological processes being the most differentially regulated (e.g., ‘innate immune response’) (Fig. 3B, Figure S5B). Comparing aCM + cond to aCM + M1 showed strongly upregulated gene clusters for aCM + M1 (Genes: 1,810 (NC-030), 1,154 (NC-059) (Fig. 3C, Figure S5B). GO analysis revealed that the 10 most highly differentially regulated processes included antigen processing and presentation, response to chemokines, and negative regulation of immune response. Further, MHC complex genes stood out as being upregulated in aCM + M1 for cell compartment and molecular function terms. This suggested that even though both conditions induced an inflammatory response, M1 coculture caused a stronger effect via distinct mechanisms. For example, genes in the HLA family (e.g., *HLA-C* (4.6 (NC-030), 4.2 (NC-059) log_2_-FoldChange) and *HLA-F* (6.1 (NC-030), 5.5 (NC-059) log_2_-FoldChange) were amongst the most highly upregulated. *A2M* was also significantly upregulated (1.1 (NC-030), 1.3 (NC-059) log_2_-FoldChange). This points at M1 coculture influencing a broader transcription profile sufficient to induce electrophysiological remodelling.

Additionally, GO meta-analysis was performed to compare the hiPSC RNA-seq data to published clinical results. For this, top GO terms from the hiPSC in vitro model were compared to all GO terms identified as significant in the CATCH ME trial[17] in sinus rhythm (N = 55) vs. paroxysmal (N = 39) AF heart tissue isolated from patients without heart failure. Interestingly, all CATCH ME trial GO terms were found as significant in the hiPSC model, e.g., MHC protein complex (NC-030: P = 6.6e − 10, NC-059: P = 1e − 10) and antigen binding (NC-030: P = 1.4e − 7, NC-059: P = 1.8e − 8) for the aCM only vs aCM + M1 comparison (Table 1). This demonstrates a high degree of overall overlap between patient tissues and hiPSC model, validating the latter as representative of paroxysmal AF clinical phenotypes.

**Table 1:** GO meta-analysis shows significant overlap between identified GOs in clinical trial data of sinus rhythm (N = 55) versus paroxysmal AF patients (N = 39) with no heart failure (CATCH ME trial) and hiPSC model

	**aCM only vs. aCM + M1 (P-Value)**	**GO Rank**	**aCM + cond vs. aCM + M1 (P-Value)**	**GO Rank**
**Cell Compartment (Upregulated)**				
MHC protein complex	6.6065e-10 (NC-030) (****) 1e-10 (NC-059) (****)	4th 5th	3.1168e-10 (NC-030) (****) 5.2988e-09 (NC-059) (****)	1st 1st
Lumenal side of membrane	3.8750e-09 (NC-030) (****) 1e-10 (NC059) (****)	1st 1st	6.7243e-09 (NC-030) (****) 3.7714e-08 (NC-059) (****)	4th 2nd
**Molecular Function (Upregulated)**				
CXCR chemokine receptor binding	0.0027 (NC-030) (***) 3.5060e-05 (NC-059) (****)	24th 15th	0.0032 (NC-030) (***) 0.0014 (NC-059) (***)	88th 20th
Antigen binding	1.4213e-07 (NC-030) (****) 1.8158e-08 (NC-059) (****)	3rd 3rd	1.0922e-06 (NC-030) (****) 3.1452e-08 (NC-059) (****)	2nd 3rd
**Molecular Function (Downregulated)**				
Gated channel activity	0.046 (NC-030) (*)	126th		
	**Overlap Analysis (Binomial test, p = 0.05)**			
	9 of 10 (1.865E-11, ****)		8 of 10 (1.6051E-9, ****)	

Identified GOs are among highest ranking GOs in the hiPSC model (ranked by NES)

In summary, M1 coculture caused upregulation of inflammation-related transcription in aCM and resulted in a larger number of upregulated genes compared to M1 supernatant-treated aCM. Overall, macrophages acted as activators of pathways in aCM which, among others, regulate immune cell communication, receptor formation (e.g., *MHC*), as well as inflammation regulation (e.g., *A2M*). The resulting transcriptome was correlated to a paroxysmal AF phenotype.

### Anti-inflammatory compounds alleviate inflammation-induced electrophysiological phenotypes in aCM

**Fig. 4:**
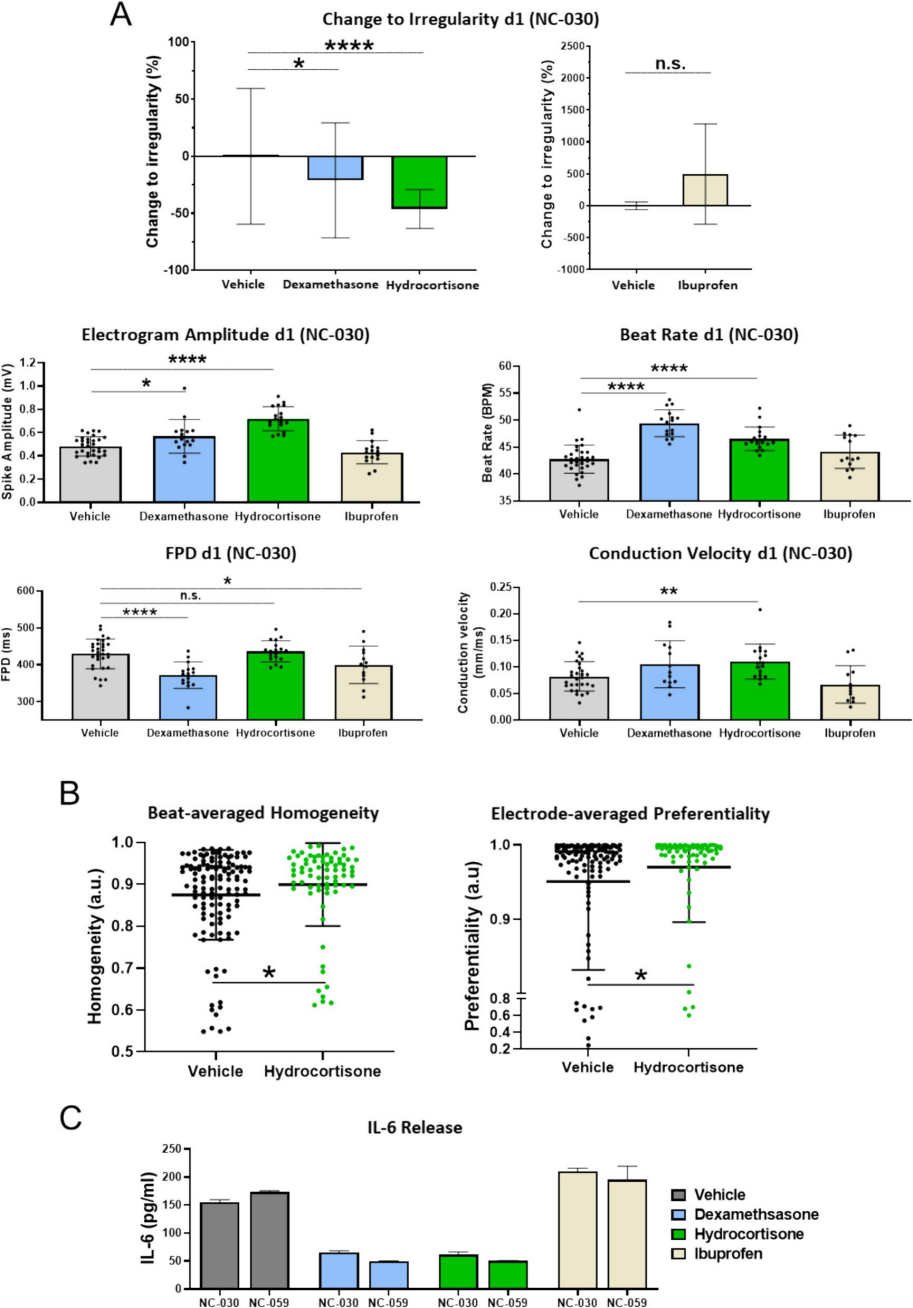
Glucocorticoids suppressed inflammation-caused arrhythmia and restored conduction homogeneity. **A** Bar graphs showing percent change compared to vehicle in beat irregularity for NC-030 aCM + M1 cocultures on d1 after activation and dexamethasone, hydrocortisone, and ibuprofen (all 10 µM) treatment, with vehicle average taken as baseline. Bar graphs showing electrogram amplitude, beat rate, FPD, and conduction velocity for NC-030 cocultures for the same conditions. **B** Conduction analysis showing scatter plot of beat-averaged homogeneity for aCM + M1 vehicle and aCM + M1 + Hydrocortisone (10 µM) on d1 after activation (Mann–Whitney test). Box plot of electrode-average preferentiality for the same conditions (Mann–Whitney test). **C** Bar graphs showing the effects of dexamethasone, hydrocortisone, and ibuprofen on IL-6 secretion in supernatants of NC-030 and NC-059 M1 macrophage monocultures 1 day after activation

Considering the observation that aCM beat rate became irregular due to inflammation, we aimed to investigate whether anti-inflammatory medication would prevent this. Intervention using glucocorticoids (dexamethasone and hydrocortisone) led to significant dose-dependent reduction in beat irregularity in NC-030 aCM + M1 cocultures compared to vehicle (irregularity average decreased 46.2% ± 17 for hydrocortisone (P < 0.0001), 21.1% ± 50.3 for dexamethasone (P < 0.05) compared to vehicle), while non-steroidal anti-inflammatories (NSAIDs; Ibuprofen) did not decrease beat irregularity (Fig. 4A, Figure S6A). Similar results were observed for NC-059 cells (Figure S6B). Importantly, glucocorticoids significantly increased electrogram amplitude (hydrocortisone NC-030 50% ± 22, NC-059 56% ± 53% increase) compared to vehicle (both P < 0.0001), while ibuprofen caused no significant change (Fig. 4A, Figure S6C). Glucocorticoids also reversed the prior reduction of electrogram amplitude from M1 coculture. Similarly, the reduced conduction velocity was restored through hydrocortisone, significantly increasing it by 38% ± 40 for NC-030 and 34% ± 44 for NC-059 compared to vehicle (Fig. 4A, Figure S6C). All compounds were tested for arrhythmogenicity on aCM monocultures, with none showing significant influence on beat rate regularity (Figure S6D).

Conduction analysis was performed to investigate whether hydrocortisone addition also influenced conduction homogeneity and preferentiality. The beat-averaged homogeneity increased significantly after treatment with hydrocortisone (0.87 a.u. aCM + M1 + vehicle, 0.9 a.u aCM + M1 + Hydrocortisone (10 µM)), both average, P < 0.05, Fig. 4B). Electrode-averaged preferentiality also showed significant increase in the hydrocortisone-treated condition (average 0.97 a.u) compared to the vehicle (average 0.95 a.u, P < 0.05, Fig. 4B).

IL-6 cytokine secretion by M1 was significantly inhibited following dexamethasone and hydrocortisone, but not ibuprofen, treatment (P < 0.0001; Fig. 4C), acting as a representative marker for the inhibition of the pro-inflammatory M1 subtype through glucocorticoids. It was hypothesised that the positive effects of glucocorticoids on aCM electrophysiology and conduction homogeneity are elicited, at least in part, through suppression of M1 pro-inflammatory subtype.

**Fig. 5:**
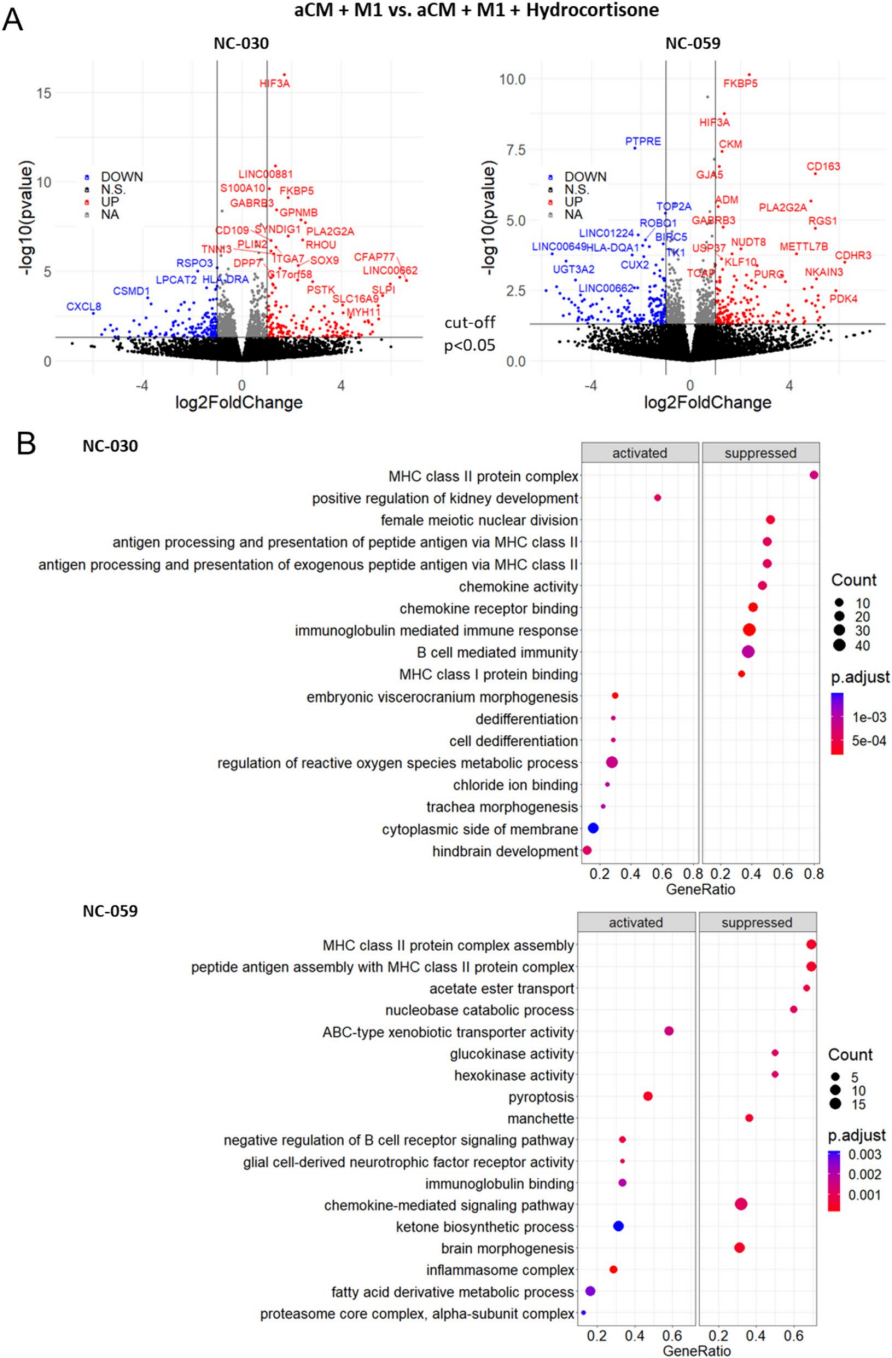
Hydrocortisone-inhibited inflammation-related gene expression in aCM. **A** Volcano plots of RNA sequencing data for NC-030 and NC-059 aCM comparing hydrocortisone-treated aCM + M1 vs. untreated aCM + M1 cocultures (see Figure S6 for NC-030 including *CKM*). Volcano plots show genes significantly (p < 0.05) changed as up (fold change > 1, red) or down regulated (fold change < -1, blue). **B** Gene ontology analysis of RNA-seq data showing biological processes activated or suppressed in NC-030 and NC-059 aCM isolated from aCM + M1 cocultures treated with 10 µM hydrocortisone vs. untreated cocultures

Transcriptomic analysis showed closer clustering between hydrocortisone-treated and untreated aCM + M1 cocultures in PCA analysis when compared to aCM only (Figure S6E). This indicated that while electrical perturbances were alleviated by glucocorticoids, drug treatment did not reverse all coculture related effects. Indeed, direct comparison of hydrocortisone-treated to untreated aCM + M1 coculture revealed fewer significant gene expression changes compared to aCM only vs aCM + M1, aCM only vs aCM + cond or aCM + cond vs aCM + M1 (Fig. 3B,C and Figure S5B). For NC-030, 469 genes were down- and 491 upregulated, while for NC-059 418, genes were down- and 455 genes upregulated (Fig. 5A, Figure S6F). Genes in both lines, whose expression was most differentially upregulated included *CKM* (2.0, 1.3 log_2_-FoldChange NC-030, NC-059 respectively) a catalyser of ATP phosphate transport, related to cardiomyocyte maturation[23], *HIF3A* (1.7, 1.4) a gene responsible for reacting to low oxygen conditions, *FKBP5* (1.9, 2.4) a gene involved in immunoregulation and protein trafficking, including intracellular trafficking of steroid hormone receptors, and *PLA2G2A* (2.5, 4.9;) a phospholipase involved in inflammation response[24], which remodels cellular membranes and is involved in pathogen clearance[25]. Genes that were significantly downregulated in both lines were *A2M* (− 0.4, − 0.5; log_2_-FoldChange NC-030, NC-059 respectively), *CXCL8* (− 5.9, − 2.2) the gene of chemokine and neutrophile attractant IL-8, *TOP2A* (− 0.6, − 1.0) a DNA topoisomerase controlling topologic states of DNA during transcription, *HLA-DPA1* (− 1.2, − 1.0) a gene part of the MHC class II involved in presenting peptides to immune cells and BIRC5 (− 0.72, − 1.1) known as survivin, which protects cells from apoptosis. The differentially expressed genes point at the aCM facing a lessened state of emergency from the hydrocortisone-treated M1. The reduced expressions of *A2M*, *CXCL8* and *HLA-DPA1*, suggest deemphasized cytokine clearance and deprioritized attraction and activation of immune cells, while the reduced expression of *BIRC5* shows a lessened need for compensatory reduction of apoptosis. Interestingly, the decreased expression of *TOP2A* might point at inflammation and its suppression altering DNA topologically. The upregulated genes support this shift, with *CKM* showing an increase in metabolism and maturation, which could be connected to reduced oxygen levels (*HIF3A*).

Gene ontology analysis revealed that addition of hydrocortisone most prominently reduced inflammation-related biological functions in aCM, notably suppressing MHC related processes (Fig. 5B). MHC class II protein complex and assembly, as well as antigen presentation and processing, chemokine binding and activity were among the highest suppressed biological functions. For NC-059 ABC-type xenobiotic transporter activity, a steroid exporting ATP-dependent transporter, was among the highest upregulated biological functions. The transporter is known to be upregulated by hydrocortisone treatment[26].

At the concentrations tested in this study, anti-inflammatories did not significantly affect M1 viability after 4 days of continuous treatment (Figure S7A,B). M1 cell identity was also unaffected, as cells retained CD68 and CD14 macrophage marker expression (Figure S7C,D). This confirmed that compound effects were due to suppressing the inflammatory activity of the M1 and not due to cytotoxicity or dedifferentiation.

In summary, glucocorticoid treatment was able to reverse inflammation-induced arrhythmic effects and restore aCM electrophysiology, including electrogram amplitude and conduction homogeneity. RNA-seq revealed transcription changes, due to inhibiting M1-caused inflammation, with especially MHC II related genes being suppressed. Of note, glucocorticoid treatment was utilized in this study not primarily as a potential therapy for AF, but rather to reverse M1 inflammatory effects and provide mechanistic confirmation for the aCM phenotypes induced by M1.

### Genes critical for cardiac function are differentially expressed in aCM due to M1 macrophage-mediated inflammation

To further investigate the mechanism of inflammation-caused arrhythmia, all genes that were either significantly up or downregulated when comparing aCM + M1 to aCM only and recovered in the comparison of aCM + M1 + hydrocortisone to untreated aCM + M1, were analysed. These genes were assumed to be mechanistically involved in the emergence of electrophysiological abnormalities in aCM + M1. A table with all 31 genes, showing significant correlation between both hiPSC lines (R^2^ = 0.89, P < 0.0001, Figure S7F) is shown in supplemental information (Table S1).

**Fig. 6:**
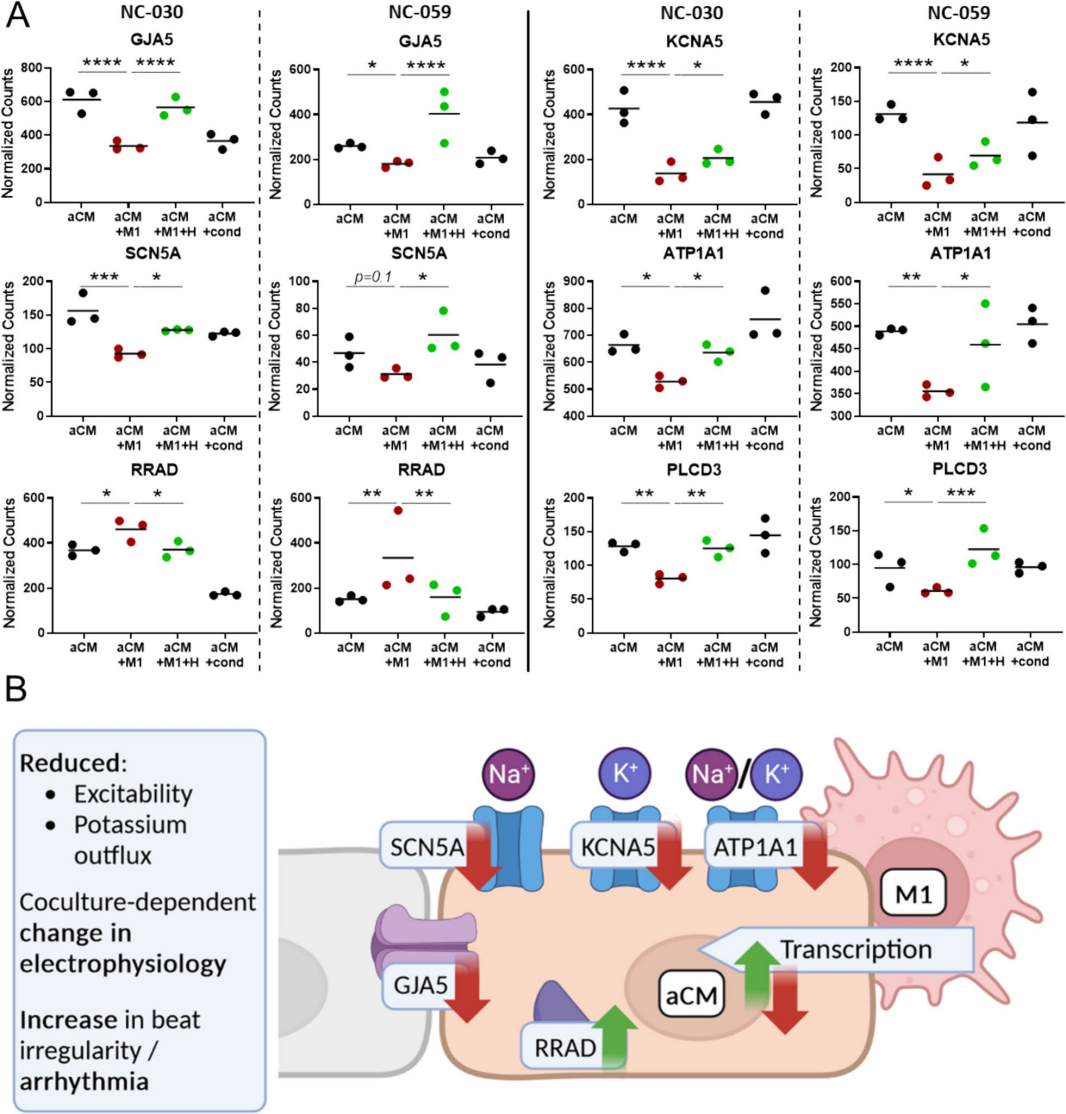
Cardiac ion channel and electrophysiology related genes were differentially affected by M1-mediated inflammation. **A** Dot plots showing the RNA-seq normalized counts for individual genes in NC-030 and NC-059. The conditions shown include aCM only, aCM + M1, aCM + M1 + 10 µM hydrocortisone (+ H) and aCM + cond. **B** Schematic representation of gene expression changes due to M1 coculture that could explain the mechanism of arrhythmia induction post M1 activation

Out of these genes, those known to affect cardiac electrophysiology (e.g., ion channels) were further analysed, highlighting significant downregulation of sodium and potassium ion channel-related genes (*SCN5A* (− 0.75, − 0.57), *KCNA5* (− 1.62, − 1.68)*, ATP1A1* (− 0.33, − 0.46); log_2_-FoldChange, NC-030, NC-059 respectively)) in aCM + M1 condition compared to aCM only. Specifically, *SCN5A* encodes the sodium voltage-gated channel α-subunit, responsible for the sodium upstroke of the action potential (*I*_Na_), *KCNA5* encodes the potassium channel α-subunit KV1.5, which forms the voltage-gated atrial-specific delayed rectifier potassium current I_kur_, and *ATP1A1* encodes the Na^+^/K^+^ Transporting ATPase, maintaining the cellular electrochemical gradient of Na^+^ and K^+^ ions. This points at reduction in I_Na_ and I_kur_ currents in the M1 coculture condition, correlating well with reduced excitation amplitude[27] and beat irregularity[28] observed in literature and during electrophysiological measurements in this study. Hydrocortisone restored gene expression to levels observed in the aCM only condition, corroborating the role of these genes in observed phenotypes. Interestingly, no such effect on ion channels was seen in the aCM samples treated with conditioned medium (Fig. 6A). *RRAD,* a calcium channel regulator involved in the suppression of voltage-gated L-type Ca^2+^ currents (*I*_CaL_) [29], was upregulated in M1 coculture and subsequently restored after hydrocortisone addition (Fig. 6A). Further, cytosolic Ca^2+^ and protein kinase C related gene *PLCD3,* known to be involved with Ca^2+^ release from intracellular stores, as well as cardiomyocyte survival[30], was downregulated in M1 coculture and restored after hydrocortisone addition, while unaffected by conditioned medium addition (Fig. 6A). Finally, RNA expression of *GJA5* (forming Cx40 proteins) was highly reduced in the M1 coculture condition, with hydrocortisone alleviating the effect (Fig. 6A), suggesting implication of this atrial-specific gap junction in aCM pro-arrhythmia. *GJA1* expression (forming Cx43 proteins in all cardiomyocyte sub-types) was unaffected by M1 coculture and hydrocortisone addition, as shown by RNA-seq (Figure S7G) and qPCR analysis (Figure S7H). Cx43 expressed by M1 was also investigated as possibly connected to observed irregularity. M1 macrophages expressed Cx43 in hydrocortisone-treated coculture with aCM (Figure S7E). qPCR analysis of the Cx43 gene GJA1 revealed activation of M0 to M1 decreased GJA1 expression (Figure S7H). M1 did not have increased GJA1 expression in coculture compared to monoculture (Figure S7I). Additionally, hydrocortisone addition did not reverse this effect, but further decreased GJA1 expression in M1 (Figure S7J). Therefore, macrophage expression of GJA1 did not appear correlated to irregularity emergence and rescue.

In summary, M1-caused inflammation and its suppression resulted in gene expression changes related to cardiac electrophysiology. The presented findings point towards the emergence of aCM arrhythmia plausibly being caused by reduced excitability, shown by a decrease of *SCN5A* transcription and electrogram amplitude. Further, a loss of potassium outflux related to *ATP1A1* and atrial specific *KCNA5,* as well as reduced *GJA5* expression, with a congruently observed reduction in conduction velocity, present a possible explanation of the pro-arrhythmic beat irregularity effects of aCM + M1 coculture. These effects could conceivably be further influenced through yet undefined mechanisms (*RRAD*, Fig. 6B). The reversal of these effects through anti-inflammatories, both in functional measurements and transcription, strongly supports macrophage-induced inflammation as the direct instigator of observed atrial perturbances.

## Discussion

This study established a novel coculture system for aCM and M1. Through the coculture, a causal relationship between M1 activation and the development of subsequent atrial arrhythmia-like irregularities was shown. As M1 conditioned medium showed no similar effect, irregularities depended on direct cell–cell contact of M1 and aCM. Transcription analysis revealed that macrophage mediated inflammation resulted in downregulation of various cardiac electrophysiology genes. The identified transcription changes were correlated to clinical AF phenotypes. Changes in gene expression may have contributed to the occurrence of electrophysiological perturbances, which will require further study.

Despite its limitations, iPSC-based modelling allowed to identify M1 coculture as a possible cause of arising arrhythmia-like phenotypes, thereby presenting the first evidence of human immune cells being the initiator of AF-related abnormalities. Regarding the question of inflammation as cause or effect of AF, this study offers evidence towards a causative role. Related to this, COVID-19, a systemic inflammatory disease elicited by SARS-CoV-2 virus infection, resulted in unexpectedly high prevalence of arrhythmias, particularly AF[31]. This provides additional evidence that inflammation can be an initiator of AF.

Further, the study presents a new model of a self-emerging AF-like phenotype. AF research in animals and hiPSC has so far depended on inducing the disease phenotype through externally introduced burst pacing[5, 15, 16, 32, 33]. The model presented here might therefore offer insight into a new pathophysiological mode of action, previously not accessible through rapid burst pacing. The suggested mechanisms found through this model, might be able to offer new therapeutic avenues, including targeted treatment on cardiac macrophages to counteract the proposed inflammation-caused AF phenotype. Prior publications have shown a positive influence of macrophages on cardiac electrophysiology, facilitating high frequency conduction in the atrioventricular node and reducing the occurrence of ventricular fibrillation[14, 34]. The findings presented here further support these results, showcasing an additional impact of of pro-inflammatory M1 on cardiac arrhythmia after being activated. Importantly, clinical trial data using paroxysmal AF patient transcriptomes show a correlation with the hiPSC aCM + M1 model, highlighting the clinical relevance of the in vitro inflammation-induced model.

For this study, glucocorticoids (dexamethasone and hydrocortisone) and NSAIDs (ibuprofen) were chosen as among the most widely utilized anti-inflammatory agents[35, 36] to help further elucidate the mechanisms of M1-induced aCM irregularities. The pronounced difference of their effectiveness in this model could point to a specific mode of action that causes inflammation-induced phenotypes and further presents the aCM + M1 model as a new tool for AF treatment discoveries. NSAIDs are cyclo-oxygenase inhibitors acting on a specific pathway, while glucocorticoids influence a wide range of processes including inhibiting pro-inflammatory cytokine secretion[37] and nitric oxide synthesis in macrophages[38]. The mitigated aCM abnormality occurrence through glucocorticoid treatment is presumed to be due to its effect on inhibiting activation of macrophages, effectively reducing the M1-caused inflammation, as shown by reduced IL-6 secretion. Importantly, it could be seen that glucocorticoids did not have a strong direct effect on aCM themselves, as shown by them not affecting aCM electrophysiology in monoculture, further supporting that their positive influence on aCM phenotypes arises from glucocorticoids acting directly on M1. Interestingly, some clinical trials have shown a positive effect of anti-inflammatory drugs in reducing fibrillation[18] and occurrence of post-operative AF (POAF)[7, 19, 20]. While large, randomized placebo controlled trials are lacking and glucocorticoids or other anti-inflammatories are not mainstay therapy for AF, evidence is mounting that the immune aspect of the disease should not be ignored.

Overall, the data presented here suggest macrophages are a critical factor in inflammation-related changes in atrial cell electrophysiology. The effect of direct coculture compared to supernatant-treated condition is evident. Cytokines released by macrophages are known to have timing dependent effects[39]. Nevertheless, all recorded time points of aCM + cond, including continuous exposures (> 24 h) to supernatant still did not result in beat irregularity, further supporting conditioned supernatants as not being pro-arrhythmic. The presented results suggest that the mechanism of M1-mediated effects on aCM includes factors beyond secreted cytokines, supporting the hypothesis that the direct interaction between macrophages and atrial myocytes is critical for the observed pro-arrhythmic effects.

Besides the direct correlation to inflammation-related expression, the transcription profiles pointed towards changes in expression of cardiac electrophysiology genes. Notable is sodium channel gene *SCN5A*, responsible for the *I*_Na_ sodium spike, i.e., electrogram amplitude. Reduction of *SCN5A* was observed in the M1 coculture, which was reversed through glucocorticoid treatment. Importantly this correlated to functional readouts in the MEA, which showed highly significant reductions of electrogram amplitude in M1 coculture, also reversed through hydrocortisone. Reduced expression of *SCN5A* could therefore be related to a loss of excitability in the aCM and lead to increased irregularity. Reduction in *SCN5A* expression has for example also been observed in sepsis-related AF mouse models[32]. Further, lowered expression of the atrial specific potassium ion channel *KCNA5* was seen in M1 coculture, also fully prevented by glucocorticoids. Reduction in this gene responsible for *I*_Kur_ could plausibly be related to the MEA findings of lower beat rate and prolonged FPD. Additionally, all of these effects could be exacerbated through the reduced expression of atrial-specific gap junctions (*GJA5,* transcribing Cx40). Importantly, conduction velocity was lower in M1 coculture and anti-inflammatory treatment alleviated this effect. GJA1/Cx43 was confirmed to be expressed in macrophages as previously reported[14], but did not show a mechanistic correlation to the observed electrophysiological perturbations.

Many of these genes (e.g., *SCN5A*[40], *KCNA5*[41], and *GJA5*[42]) have previously been related to familial AF. Specifically, *GJA5*/Cx40 is known to be reduced in atrial tissues of paroxysmal and chronic AF patients and is presumed to influence AF pathogenesis. Additionally, abnormal expression of Cx40, the most prevalent connexin in the atria, has been connected to both trigger formation and AF vulnerability[43]. This study found no reduction in *GJA1/*Cx43, as seen previously in human AF samples[17, 44]. RNA-seq analysis in this study revealed upregulation of the calcium channel regulator *RRAD* in the M1 coculture condition, which is notable as this gene has previously been linked to arrhythmia[45]. In particular, a gain of function *RRAD* mutation has been associated though an hiPSC model to a familial case of Brugada syndrome, a channelopathy exhibiting right bundle branch block and slowed cardiac conduction[45]. Moreover, *RRAD* was reported as significantly upregulated in paroxysmal and persistent AF patient heart tissue samples in the CATCH ME clinical trial[17].

Overall, the two hiPSC donor lines used in this study showed similar results regarding transcriptomics and functional readouts. Of note, a different effect size in irregularity for the aCM + M1 condition was observed. Patient-specific responses are not uncommon between donor lines and are likely due to underlying genetic variation[46].

A limitation of this study is the known immaturity of hiPSC-derived cells. hiPSC-derived cardiomyocytes have a more fetal-like electrophysiology and show automaticity[47, 48]. In addition, the in vitro coculture model does not recapitulate the full immune system or other aspects of in vivo physiology, which could limit the predictive value of the model. Nevertheless, hiPSC cardiomyocytes are functionally relevant human cells with correlated pathophysiological phenotypes, that offer complementary insights to other models. Another limitation of this study is that current immune cell research suggests that macrophages possess high plasticity, blurring the lines between subtypes[49]. Simple separation in M1 and M2 macrophages does not recapitulate all cell subtypes[13, 50, 51], or differences between tissue-resident and blood-derived macrophages[50]. This study chose to focus on pro-inflammatory M1 macrophages. How each of these subtypes affects cardiac electrophysiology has not been conclusively investigated and could be the subject of further research. Finally, IL-6, as a pro-inflammatory cytokine, was chosen to representatively assess macrophage activation, while not further investigating other cytokines. Despite limitations, the AF-like in vitro model lends itself well to additional research that could elucidate the effects of comorbidities, such as infection or fibrosis, on disease severity and progression.

## Conclusions

The presented study identified pro-inflammatory macrophages (M1) as a cause of arrythmia-like event induction in an atrial cardiomyocyte (aCM) and M1 coculture, using a new hiPSC-based disease model of AF without the need for additional electrical burst pacing. Further, transcriptomic and functional analysis revealed M1 to cause electrophysiological changes in aCM, including reduced conduction velocity and decreased expression of sodium and potassium channel related genes, offering a possible explanation for the mechanism of inflammation-induced AF. Glucocorticoids showed reversal of M1-induced expression changes, as well as alleviation of electrophysiological phenotypes, which correlate to clinical findings and offer further evidence towards inflammation being causative of electrophysiological abnormalities. Finally, clinical trial meta-analysis revealed highly significant correlation between our model and AF patients’ transcription profiles.

## Methods

### hiPSC culture

NC-030 hiPSC line (female, adult, from renal epithelial cells, episomal reprogramming, LUMC hiPSC core facility) and NC-059 hiPSC line (CRMi001-A, male, fetal, from CD34 + cord blood cells, episomal reprogramming, NIH Center for Regenerative Medicine (CRM)) were used. hiPSCs were seeded on Matrigel (Corning), cultured in mTeSR1 (StemCell Technologies) with 50 U/mL Penicillin/Streptomycin and passaged twice a week with Fasudil (LC Laboratories) (5 µM) supplement using a DPBS- (Life Technologies) wash and Accutase (Sigma-Aldrich).

### hiPSC derived atrial-like (aCM) and ventricular-like (vCM) differentiation and culture

aCM and vCM differentiation protocols were adapted from the proprietary vCM differentiation protocols of Ncardia. aCM and vCM were differentiated from NC-030 and NC-059 in monolayer with 74k cells per cm^2^ on Matrigel (Corning) (1:100) seeded at day -1 before differentiation. Cardiac mesoderm was induced at day 0 by switching to cardiac differentiation medium (Ncardia) supplemented with small molecules selectively activating and inhibiting Wnt pathways. Atrial subtype was induced through the addition of Retinoic Acid (RA). Medium was changed every 2–3 days and cells were dissociated using TrypLE Select (1x) (Life Technologies) at day 14 and cryopreserved in cardiac cryopreservation medium (Ncardia) supplemented with 10% DMSO (Sigma-Aldrich). For all NC-030 vCM comparisons, commercially available Ncytes (Ncardia) were used, if not specified otherwise.

Cryopreserved aCM and vCM vials were thawed in media supplemented with 10 µM Y27632 (Axon Medchem) and cultured using Pluricyte culture medium (PCM), seeded on Fibronectin (Sigma-Aldrich) diluted 1:100 in DPBS + (Life Technologies). Medium was changed every 2–3 days. For all CM assays cells were cultured for > 14 days post-cryopreservation before being used in functional assays, if not specified otherwise.

### Intracellular action potential (sharp electrode) recordings

Cells seeded on coverslips (ThermoFisher Scientific) were taken out of the cell culture incubator at 7 ± 2 days after seeding and placed in a perfusion chamber (RC-26G, Warner Instruments) under constant bath solution flow controlled by a peristaltic pump (Easy-Load II Pump, Masterflex L/S) at 2 mL/min, at 35 ± 2 C. Oxygenated modified normal Tyrode’s solution (NaCl 140 mM, KCl 5.5 mM, HEPES 10 mM, MgCl2 1 mM, Glucose 10 mM, CaCl_2_ 1.8 mM) was used as bath solution. Temperature was controlled via flow-through (SH-27B, Warner Instruments) and chamber heaters (PH1, Warner Instruments), using a two-channel controller (TC-344B; Warner Instruments). Cells usually sitting in large multi-layered clusters were impaled with microelectrodes achieving a resistance of 15–20 MΩ by pulled glass capillaries (Clark borosilicate with filament, OD 1.00 / ID 0.58, 100 mm, Warner Instruments) using a Sutter P-97 micropipette puller (Sutter Instrument). Microelectrodes were filled with 3 M KCl and connected to a bridge amplifier (BA-01X, NPI Electronic) via an Ag–Ag-Cl electrode. The reference electrode placed in the bath was an Ag–AgCl pellet and wire electrode (E205, Ø 1.0 mm, Harvard Apparatus). Micropositioning of the electrode was achieved using a TSC Sensapex micromanipulator (Oulu, Finland) and controlled using an IX70 microscope (Olympus). Recordings were acquired at 50 kHz and filtered at 10 kHz by a LabView (National Instruments) custom-made script.

### Monocyte differentiation, culture, and maturation to macrophages

iPSC differentiation protocol towards a monocyte tissue-resident lineage was adapted from Gutbier et. al[52]. Dissociated NC-030 and NC-059 iPSC were transferred to AggreWells 800 wells (StemCell Technologies) and cultured as spheroids from day 0 to day 4 in mTesR supplemented with 50 ng/mL BMP4 (R&D systems), 50 ng/mL VEGF (R&D systems), 20 ng/mL SCF (Miltenyi Biotec). Spheroids were subsequently moved to 75 cm^2^ cell culture flasks (Corning) coated with Matrigel (1:100) and kept in culture with weekly medium changes using complete monocyte medium (X-Vivo 15 (Lonza) with 1% v/v GlutaMAX Supplement (Gibco), 50 U/mL Penicillin/Streptomycin, 0.05 mM 2-Mercaptoethanol (Gibco) supplemented with 100 ng/mL M-CSF (Gibco) and 25 ng/mL IL-3 (Gibco). Monocytes were harvested from cell culture supernatants every week 4 weeks after seeding into T75 flasks. Monocytes were cultured in PCM, unless stated otherwise.

Maturation of monocytes towards M1 macrophages was performed by supplementing culture medium with 20 ng/mL GM-CSF (Gibco) for 6 days, with medium being refreshed at d3. Matured M1 macrophages were activated by adding 100 ng/mL IFN-γ (Peprotech) and 50 ng/mL LPS (InvivoGen) at d6 for 20h, followed by a change of medium with 100 ng/mL LPS added for 4h. Harvested monocytes and macrophages were cultured on uncoated polystyrene, unless otherwise specified.

### Cell fixation

Adherent cell cultures were fixed using 4% PFA for 15 min at RT after being washed once with DPBS- and rinsed twice more with DPBS- thereafter. Cell suspensions were fixed using Inside Stain Fix kit (Miltenyi) according to the manufacturer’s protocol. All fixations for transcription factors (COUP-TF II) were performed using Transcription Factor Buffer Set (BD Pharmingen) and Stain Buffer (BD Pharmingen) according to the manufacturer’s protocol.

### Flow cytometry

Flow cytometry was performed using a Novocyte Flow Cytometer 200 (ACEA Biosciences), with all washes and dilutions performed using FACS buffer (Ncardia). Suspended, previously fixed aCM and vCM of both lines were co-stained with antibodies for cTnT Reafinity conjugated FITC (1:10, Miltenyi) and MLC2a Reafinity conjugated APC (1:10, Miltenyi) with an incubation of 15 min at RT. 100k cells were used per sample and flow cytometry was performed at d14 and d28 after the start of differentiation. Samples were gated to isotype control (REA control FITC, REA control APC, Miltenyi). Same conditions were also co-stained for COUP-TF II (primary antibody: 1:100, R&D Systems) and cTnT (as above). Samples were incubated with the primary COUP-TF II antibody for 45 min at 4°C, in the dark, followed by incubation with the secondary antibody (APC) AffiniPure F(ab')₂ Fragment Donkey Anti-Mouse IgG (H + L), 1:500, Jackson ImmunoResearch) and conjugated cTnT antibody for 45 min at the same conditions. Samples were gated to isotype controls (Purified Mouse IgG2a, κ, (BioLegend); REA control FITC).

Live cell flow cytometry was performed for monocytes after supernatant harvesting during differentiation (> d31). Washes and dilutions were performed using FACS Buffer. 100k cell samples were triple stained using conjugated IgG1 mouse anti-human antibodies for CD45 (1:20, PE), CD11b (1:20, APC), CD14 (1:20, FITC), (all BioLegend) and incubated for 20 min at 4°C in darkness. Samples were gated to isotype controls (Mouse IgG1-FITC; PE; APC, all BioLegend).

### Brightfield Imaging

Brightfield imaging and video recording was performed using a Nikon eclipse ts100 microscope (Nikon, Japan) and a ToupCam LCMOS05100KPA Camera (ToupTek, China).

### Immunofluorescence staining (IF)

All IF imaging was performed using an ImageXpress Micro Confocal high content imager (Molecular Devices) with PFA fixed adherent cells (unless specified otherwise) using black 96 well µClear® CELLSTAR® plates (Greiner), including DAPI (1:1000, Invitrogen) staining during secondary antibody incubation. All dilutions used PBST-FBS (DPBS- (Life Technologies), Tween20 (ThermoFisher Scientific), FBS (Gibco)) and all samples were washed with PBST and blocked with PBST-FBS.

Activated M1 macrophages, matured M1 macrophages without activation factors, M0 macrophages (all d10) and naïve monocytes (d1) were stained with primary antibodies for Vimentin human anti-human REAfinity™ conjugated FITC (1:50, Miltenyi), CD14 mouse anti-human IgG1 conjugate FITC (1:100), CD11b mouse anti-human IgG1 conjugated APC (1:50), CD68 mouse anti-human (eBioY1/82A (Y1/82A)) conjugated FITC (1:200, Invitrogen) and CX3CR1 rabbit anti-human (1H14L7) (1:250, Invitrogen) and corresponding isotype controls (REA control FITC 1:50, Mouse IgG1-FITC 1:50, Mouse IgG1-APC 1:100, Mouse IgG2b-FITC 1:1600 (Invitrogen), Rabbit IgG 1:1500). Samples were stained with corresponding secondary antibodies (Goat anti-human IgG (H + L) Alexa Fluor 488 1:500 (ThermoFisher Scientific), Donkey Anti-Rabbit IgG (H + L) Alexa Fluor 594 (ThermoFisher Scientific), Donkey anti-mouse IgG (H + L) Alexa Fluor 488, Goat anti-Mouse IgG2b Alexa Fluor 647 (ThermoFisher Scientific) (all 1:200). Incubation times were as previously described.

Isogenic cocultures of aCM and activated M1 (20,000 + 5000) were stained on d10 (3 days post activation) in black 96 well plates and 96 well CytoView MEA plates (Axion BioSystems), after MEA recording, both fibronectin coated. Primary antibodies used were CD68 mouse anti-human (eBioY1/82A (Y1/82A)) conjugated FITC (1:200), CX3CR1 rabbit anti-human (1:250) and cTnT REAfinity conjugated APC (1:100, Miltenyi) and corresponding isotype controls (Mouse IgG2b-FITC 1:1600, Rabbit IgG 1:1500 and REA Control APC 1:50 (Miltenyi)). Corresponding secondary antibodies were Donkey anti-mouse IgG (H + L) Alexa Fluor 488, Donkey Anti-Rabbit IgG (H + L) Alexa Fluor 594, Goat anti-Human IgG (H + L) Alexa Fluor 647 (ThermoFisher Scientific), all diluted at 1:200 in PBST-FBS. Incubation times were as previously described.

aCM monocultures or cocultures of aCM and activated M1 or M0 macrophages (20,000 + 5,000, 8 or 10 days after seeding) were seeded on fibronectin coating 1:100 and stained with primary antibodies CD11b mouse anti-human IgG1 conjugated APC (1:50), cTnT REAfinity conjugated FITC (1:100) and Connexin 43 ZooMAb® Rabbit (Sigma-Aldrich) and corresponding isotype controls (Rabbit IgG 1:1500, Mouse IgG1-APC 1:100 and Rea Control FITC 1:50). Matching secondary antibodies Donkey Anti-Rabbit IgG (H + L) Alexa Fluor 594, Goat anti-Mouse IgG2b Alexa Fluor 647 and Goat anti-human IgG (H + L), Alexa Fluor 488 (all 1:500) were used. Incubation times were as previously described.

IF co-staining for COUP-TF II, cTnT and DAPI was performed on fixed (BD Pharmingen kit) d30 aCM and vCM (NC-030) seeded at 20,000 cells per well on fibronectin coated (1:100, in DPBS +) 96 well plates. Washed and blocked wells (PBST, PBST-FBS) were incubated with primary/conjugated antibodies (COUP-TF II 1:100, cTnT conjugated to FITC 1:100) and correlating isotype controls for 1h at RT. Samples were incubated with secondary antibodies (Goat anti-Mouse IgG1 Alexa Fluor 555, 1:500; Goat anti-human Alexa Fluor 488, 1:500) for 1h at RT, before being washed and imaged. aCM/vCM (NC-030) were also stained under same conditions for MLC2a, MLC2v. Wells were incubated with primary antibodies (MLC2a (Monoclonal Mouse Antibody [56F5] 1:500 (Synaptic Systems), MLC2v Rabbit Pab 1:150 (ProteinTech Group) and correlating isotype controls (Mouse IgG2a, k; Rabbit IgG (Invitrogen)) overnight at 4°C on a plate shaker. Samples were incubated with secondary antibodies (Goat anti-Mouse IgG1 Alexa Fluor 555 1:500 (ThermoFisher Scientific), Goat anti-human IgG (H + L), Alexa Fluor 488 1:500 (ThermoFisher Scientific); Chicken anti-Rabbit IgG Alexa Fluor 647 1:500 (ThermoFisher Scientific) for 1h at RT, before being washed and imaged.

### Phagocytosis assay

Phagocytic activity was investigated using bioparticles taken up by phagosomes[53]. Monocultures of M1 activated macrophages at 8 days post seeding, (32.000 cells per well of 96 well plate) had pHrodo Green zymosan yeast bioparticles (ThermoFisher) added (50 µg/mL) to the cell culture media and were co-stained with Hoechst dye (1:1000) (ThermoFisher). Cells were incubated for 10 min, at 37°C, and imaged under fluorescence as previously described.

### RNA extraction, cDNA synthesis, and qPCR

RNA extraction and cDNA synthesis were performed using the NucleoSpin RNA Mini kit (Machery-Nagel) and iScript™ cDNA Synthesis Kit (BioRad) according to manufacturers’ protocols, using an iQ5 thermal cycler (BioRad). qPCR was performed using SsoAdvanced Universal SYBR® Green Supermix (BioRad) and an iQ5 thermal cycler according to manufacturer’s instructions. All primers were synthesized by Integrated DNA Technologies, BV, except *IL10*, *IL6* and *IL12A* (BioRad). Fold change was normalized to a housekeeping gene (GAPDH) and reference conditions (∆∆). Macrophages from cocultures isolated through CD14 magnetic bead cell sorting (CD14 Microbeads human, MS column, MiniMACS kit, all Miltenyi) according to manufacturer’s protocol prior to RNA extraction.

### IL-6 colorimetric ELISA assay

IL-6 ELISA was performed using IL-6 Human uncoated ELISA Kit (Invitrogen). Supernatants were collected from cell culture plates, centrifuged at 400 g for 5 min and, following debris removal, stored at -80°C. d7, d8, d9 and d10 samples were sequentially collected from same wells, with medium refreshed after each removal. Colorimetric assay was performed according to manufacturer’s protocol, including control wells for all medium types. Final readouts were correlated to seeding density of each sample and adjusted to control medium values. Cell cultures were fixed with PFA, stained with DAPI and imaged (as previously described) to ascertain continued cell presence.

### MEA seeding and recording

20.000 cells, unless specified otherwise, were seeded in a droplet on electrodes of fibronectin coated (1:20) MEA plates with cell culture medium supplemented after adhesion of cells for 2 h. For all MEA recordings, a Maestro Pro (Axion BioSystems) instrument was used. Environmental conditions were maintained at 37°C, 5% CO2, with all plates equilibrated for 30 min prior to recordings. Recordings and processing were performed using Axis Navigator (Axion BioSystems), to analyze beat rate (beats per minute; BPM), conduction velocity (mm/ms), FPD (ms) and beat irregularity (%, coefficient of variation in percentage; Eq. 1).

*Equation* 1 *: Beat irregularity (%) formula*

1 $$Beat irregularity \left(\%\right)=\frac{Standard Deviation (beat rate)}{Mean (beat rate)} x 100$$

Isogenic aCM and macrophage cocultures were combined in suspension and seeded in a droplet (20,000 + 5,000 per well) in 96 well Cytoview MEA plates. Plate wells contained 8 electrodes plus a reference electrode, utilizing unipolar recording. Medium (PCM, 200 µL per well) was added according to M1 maturation/activation medium change schedule (as described before), with non-activated conditions having no added GM-CSF, LPS and IFN-γ. For coculture characterization, 5 conditions were recorded daily from d6 after seeding through d10: aCM only, aCM + M0, aCM + activation factors, aCM + M1, aCM + conditioned medium from M1 monoculture. For conditioned medium conditions, 20 µL supernatant from M1 monocultures (CellStar 12 well plate (Greiner), 500,000 cells in 1 mL PCM) was added to wells. Addition was performed 2 h prior to the first recording, with supernatant being additionally added every following day. Plates were recorded for 10 min.

### Compound treatment

Hydrocortisone, dexamethasone, ibuprofen (0.1,1,10 µM, all ThermoFisher) and vehicle (0.1% DMSO) were added to cells during MEA recordings. Cocultures were seeded and cultured as previously described and serial dilutions were added during medium changes at d6, d6 + 20h, d7 and d8 after seeding. MEA recordings were performed on d8 after seeding with plates being recorded for 40 min.

MEA recordings of drug treatments for cardiac sub-type characterization were performed using Carbamoylcholine chloride (Carbachol) at 0.1, 1, and 10 µM, in DMSO (Tocris) and vehicle (0.1% DMSO). Serially diluted compounds were added to cells 2h prior to 10 min MEA recordings.

Arrhythmia induction was performed using ivabradine hydrochloride (ThermoFisher Scientific) in DMSO at 0.3 and 1 µM, isoproterenol (ThermoFisher Scientific) in DMSO at 1,10,100, 1000 and 10000 nM and aconitine (Sigma-Aldrich) at 0.1,1 and 10 µM in water and vehicle (0.1% DMSO, water respectively). Serially diluted compounds were added 48h prior (ivabradine) or 30 min prior (isoproterenol, aconitine) to 10 min recordings.

NaV1.5 inhibition was performed using Flecainide (MedChemExpress) in DMSO at 1 and 10 µM and vehicle (0.1% DMSO). Serially diluted compounds were added 0.5h prior to 10 min recordings.

### Bulk RNA-sequencing

For bulk RNA sequencing, 4 conditions were analyzed: aCM only, aCM + M1 conditioned medium, aCM + M1 and aCM + M1 + 10 µM Hydrocortisone. All samples were seeded on fibronectin coated (1:100) 24 well Cellstar plates (Greiner) with 500,000 aCM per well and 125,000 M1 added to cocultures. Medium (PCM, 500 ul per well) was added according to M1 maturation/activation medium change schedule (as described before), with non-activated conditions having no added GM-CSF, LPS and IFN-γ. Hydrocortisone addition was performed as previously described. For conditioned medium samples, 125 ul supernatant from M1 monocultures (24 well plate, 500,000 cells/well in 0.5 mL PCM) was added to appropriate samples. Addition was performed 2 h prior to cell collection. On d8, cells were detached using TrypLe-Express (Gibco) and single cell suspensions generated. M1 cells in coculture suspensions were removed through CD14 magnetic bead cell sorting (CD14 Microbeads human, MS column, MiniMACS kit, all Miltenyi) according to manufacturer’s protocol. aCM cell populations were stored at -80°C and used for RNA extraction (as previously described). Bulk RNA-seq was performed on RNA samples by SingleCellDiscoveries. Count data was analyzed using R-studio (R-studio, PBC, Boston, USA) and Deseq2[54]. P values were analyzed using Wald test, with a *P* value < 0.05 regarded as significant. Genes were annotated for ontologies using clusterProfiler and DOSE[55, 56]. Expression correlation of fold change values was analysed using Pearson correlation.

### Homogeneity and preferentiality mapping

Conduction direction vectors were extracted from performed MEA coculture recordings. These vectors were defined as unit normalized conduction velocity vectors on each electrode for a single beat where conduction velocities were calculated by a previously defined finite difference technique[57]. Conduction directions were extracted for a single beat if at least 3 electrodes (out of 9) were activated. Missing activations were imputed with linear interpolation. Homogeneity of a beat was defined as the norm of the electrode-averaged conductions direction vectors during that beat. In cases where electrodes exhibited similar conduction directions, homogeneity value was close to the unity (e.g. 1.0 a.u.) while more complex conduction patterns were manifested by lower homogeneity values. We extracted a single homogeneity value for each experiment by averaging over all beats. Preferentially value was used to quantify the invariability of the conduction direction over an electrode: Conduction directions for a single electrode was averaged over all beats. The resulting preferentiality was close to the unity (e.g. 1.0 a.u.) if conduction direction remained similar during the recording. Preferentially values for all electrodes of one sample were averaged.

### Live-dead cell staining

Live-dead staining was performed on activated M1 macrophages 10 days after seeding, seeded at 50,000 cells in uncoated black 96 well plates. Cells were treated with compounds (all [10 µM]) or vehicle (0.1% DMSO) continuously starting at d6. Cells were co-stained with TO-PRO™-3 Iodide and SYTO™ 14 (both Invitrogen) according to manufacturer’s instructions and imaged under fluorescence as previously described. Positive controls were treated with 70% EtOH for 5 min. Viability was calculated as percentage of cells expressing SYTO™ 14, while having no expression of TO-PRO™-3 Iodide. Images were analyzed and percentages calculated by ImageXpress MetaXpress (Molecular Devices).

### *Meta*-analysis of clinical trial RNA sequencing data

Published gene lists and gene ontologies from the CATCH ME trial in Zeemering et.al, Heart Rhythm, 2022[17] were compared to RNA sequencing performed in this study. Expression values from NC030 and NC059 lines were averaged and genes with zero-value fold change removed. Genes not present in the clinical trial gene lists and RNA sequencing set from this study were removed. Enrichment for GOs in the hiPSC RNA-seq was tested using binomial test (*P* = 0.05).

### Statistical analysis

All datasets were checked for normality and lognormality. Student t-test was used for statistical analysis of independent, normally distributed data populations, if not stated otherwise. Mann–Whitney test was used for all not normally distributed irregularity related analysis. One-way ANOVA was used for grouped analysis, using non-parametric analysis for not normally distributed data. A *P* value ≤ 0.05 was regarded as significant. All variance data is shown as standard deviation, unless stated otherwise. Data entry and calculations were performed using Excel (Microsoft). Graphical and statistical analysis was performed using GraphPad Prism 8 (Graphpad Holdings, LLC).

## Supplementary Information

**Supplementary material 1**

**Supplementary material 2**

## Data Availability

The data of this study are available from the corresponding author upon reasonable request. RNA-seq data are available at the Gene Expression Omnibus database under accession number GSE236870.

## Abbreviations

aCM: Atrial cardiomyocytes
vCM: Ventricular cardiomyocytes
hiPSC: Human induced pluripotent stem cells
IF: Immunofluorescence
