# Supplementary material for: Immune response caused by M1 macrophages elicits atrial fibrillation-like phenotypes in coculture model with isogenic hiPSC-derived cardiomyocytes
Source: Stem Cell Res Ther. 2024 Sep 4;15:280. doi: 10.1186/s13287-024-03814-0 (PMC11373469; doi:10.1186/s13287-024-03814-0)
Supplement: Supplementary file 2 — Supplementary material 2 [file 13287_2024_3814_MOESM2_ESM.docx]

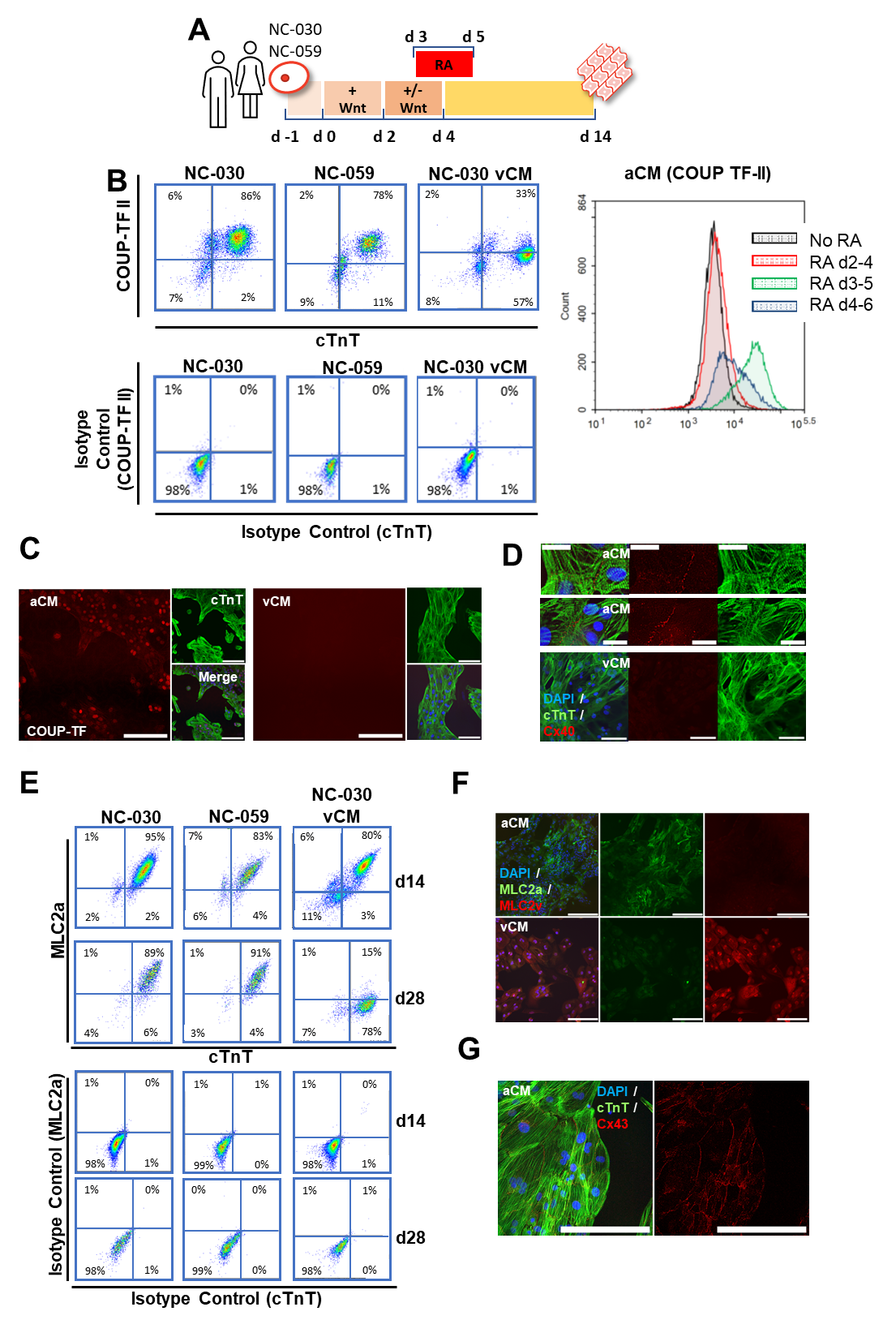
Figure S1

**Figure S1: Atrial differentiation yielded atrial-like cardiomyocytes, expressing subtype specific biomarkers**

**A)** Protocol schematic of aCM differentiation from hiPSC for 2 healthy donor lines (NC-030, female; NC-059, male), showing timepoints for selective Wnt de-/activation and Retinoic acid (RA) addition. **B)** Flow cytometry images showing atrial-specific marker COUP-TF II(58) and cTnT double staining and isotype controls in aCMs (NC-030, NC-059) and vCM (NC-030) on day 14 of differentiation. aCMs showed highly upregulated expression of COUP-TF-II (~80%) compared to absence in vCM, while both subtypes showed high cardiomyocyte purity (cTnT+ ~90%). Single staining for COUP-TF II in NC-030 showing marker expression dependent on Retinoic acid (RA) time window. **C)** IF images of NC-030 aCM and vCM staining on day 28 of differentiation of culture for COUP-TF II, cTnT and DAPI showing nuclear co-localization of COUP-TF II and DAPI for cTnT+ aCM and absence of COUP-TF II in vCM. **D)** IF images of cTnT, DAPI and atrial specific Cx40 staining in aCM and vCM (NC-030, 28 days post differentiation) showing intercellular detection of Cx40 for aCM and absence in vCM (scale bar 20µm aCM; 100µm vCM). **E)** Flow cytometry images of MLC2a and cTnT double staining for aCM (NC-030, NC-059) and vCM (NC-030) at d14 and d28 post differentiation, showing subtype-specifc retained MLC2a(59) expression by aCM over time and loss of MLC2a in vCM, as well as isotype control images for MLC2a and cTnT double staining. **F)** IF images of aCM and vCM on day 28 post differentiation stained for MLC2a, MLC2v and DAPI, showing presence of MLC2a and absence of MLC2v in aCM, with the reverse for vCM (scale bar 200µm). **G)** IF images of aCM day 28 post differentiation stained for cTnT, Cx43 and DAPI, showing presence of Cx43 at the outline of the cell membrane (scale bar 100µm).

Abbreviations: atrial cardiomyocytes (aCM), ventricular cardiomyocytes (vCM), retinoic acid (RA), Immunofluorescence (IF)

**
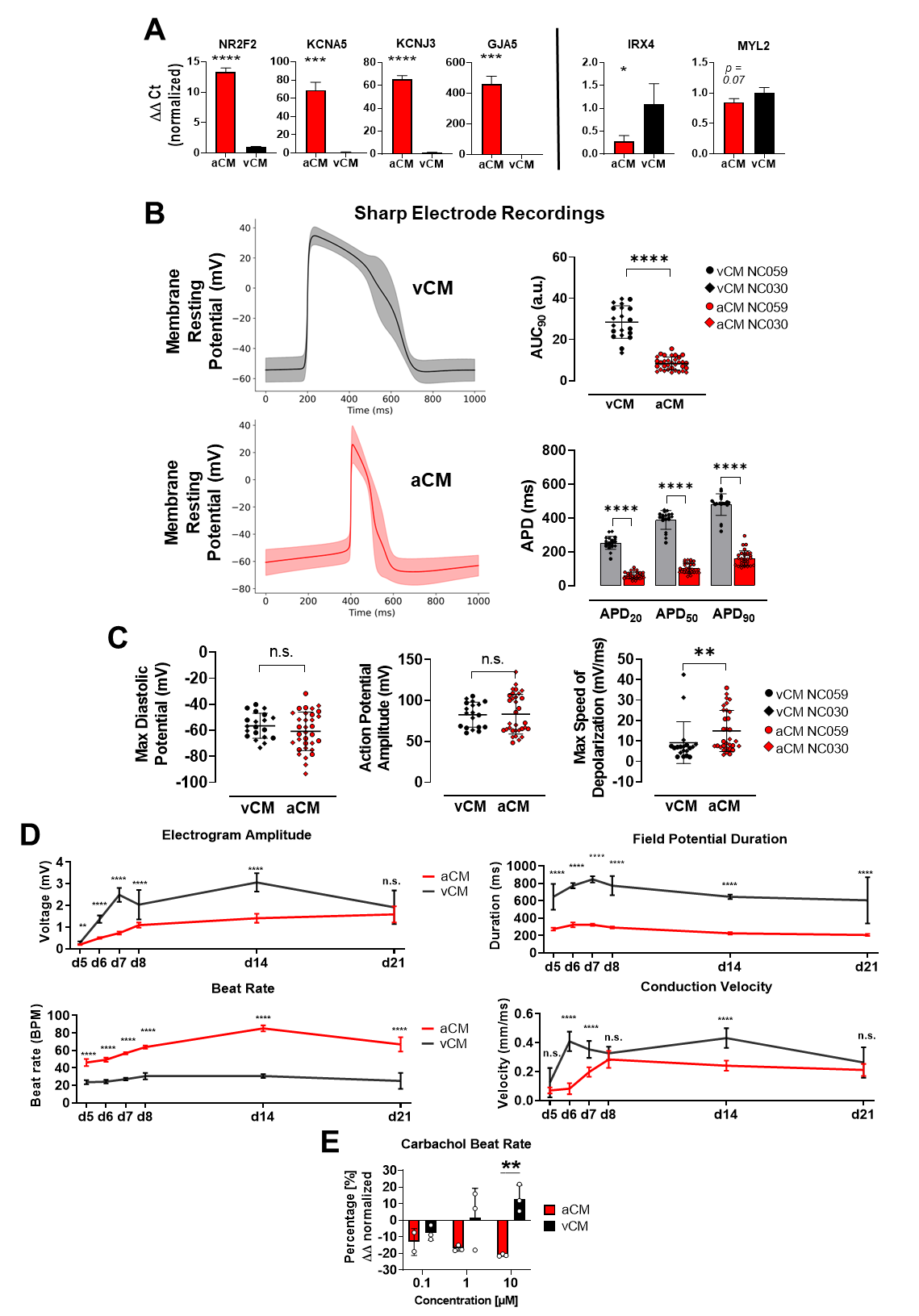
**Figure S2

**Figure S2: Atrial differentiation yielded atrial-like cardiomyocytes with subtype specific electrophysiology**

**A)** RT-qPCR expression levels for atrial subtype markers(58,60) *NR2F2, KCNA5*, *KCNJ3*, *GJA5* and ventricular subtype markers(60) *IRX4*, *MYL2* for aCM and vCM 28 days post differentiation, values ΔΔ Ct normalized to GAPDH and vCM expression levels. **B)** Sharp electrode recordings of aCM and vCM showing superimposed averaged traces of aCM (traces n=311) and vCM (n=301) (both lines) and graphs showing area-under-curve_90_ (AUC_90_) and action potential duration (APD) for each subtype, including both lines, with highly significant differences between types with shorter APDs and lower AUC_90_ for aCM. **C)** Sharp electrode recordings of aCM and vCM showing Max Diastolic Potential, Action Potential Amplitude and Maximum Speed of Depolarization for both lines, with significant difference for speed of depolarization between vCM and aCM. **D)** MEA data in NC-030 aCM and vCM showing electrogram amplitude, Beat Rate, Field Potential Duration (FPD) and Conduction velocity for day 5 through day 21 post seeding (i.e., day 19 through 35 post differentiation). Subtypes were significantly different at individual timepoints (based on multiple t-tests). Error bars 95% CI **E)** Compound treatments in aCM and vCM (NC-030) using Carbachol. Carbachol showed atrial subtype-specific reduction in beat rate, with significant changes compared to vCM for 10 µM. Carbachol was normalized to vehicle and baseline. Multiple t-test was used for subtype comparisons.

Abbreviations: atrial cardiomyocytes (aCM), ventricular cardiomyocytes (vCM), multi electrode array (MEA)

**
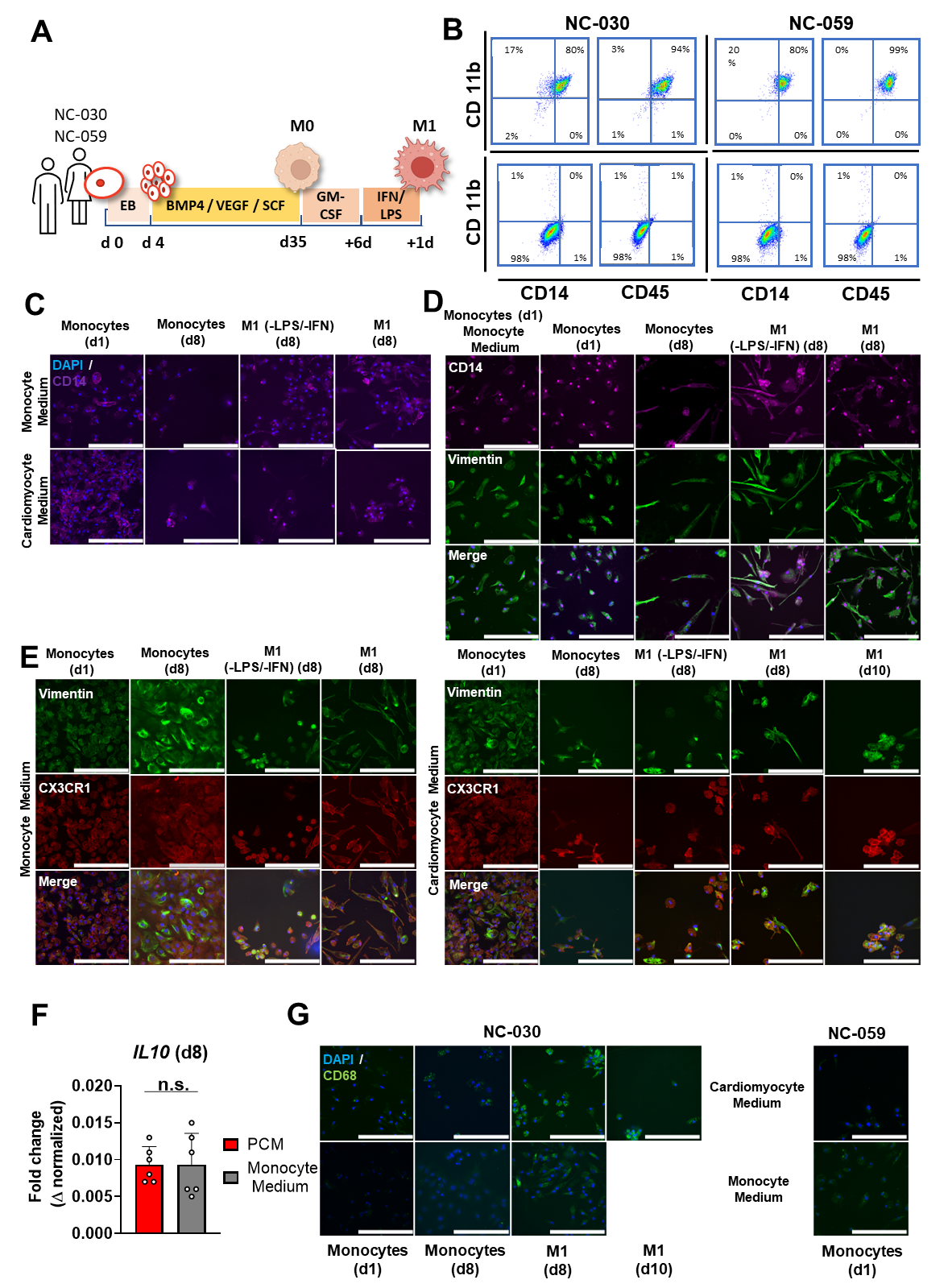
**Figure S3

**Figure S3: Macrophage differentiation and activation resulted in M1 subtype biomarker expression**

**A)** Schematic of protocol adapted from Gutbier et. al(52) for monocyte differentiation and M1 maturation from hiPSC for 2 donor lines (NC-030, female; NC-059, male), showing timepoints for embryoid body formation (EB), monocyte differentiation, harvest (d35), maturation into M1 macrophages (+6d) and activation. **B)** Flow cytometry images of hiPSC-derived monocytes of both lines at harvest (d0), showing leukocyte and monocyte/macrophage marker(61) expression of CD14 and CD45, both co-stained with CD11b and indicating a triple positive population of ~80% monocytes for both lines and corresponding isotype controls. **C)** IF images of NC-030 DAPI and CD14 co-staining, showing continued marker expression in both monocyte and cardiomyocyte medium (PCM), as well as throughout macrophage maturation and different stages of activation (scale bar 100µm). **D)** IF images of NC-059 stained for Vimentin, CD14 and DAPI showing continued marker expression in both monocyte and Cardiomyocyte medium (PCM), as well as throughout maturation and different stages of activation (Scale bar 100µm). **E)** IF images of NC-030 stained for Vimentin, DAPI and tissue-resident lineage marker CX3CR1(14,62,63), showing persistent expression of Vimentin and tissue-residence niche marker CX3CR1 throughout macrophage maturation and activation, as well as in culture with monocyte medium and cardiomyocyte medium (PCM; scale bar 100µm). **F)** *qPCR expression levels for IL10 at day 8 of culture in PCM or monocyte medium. Expression given as fold-change normalized to GAPDH levels.* **G)** IF images of DAPI and CD68 staining showing absence of the activation marker CD68(64) in monocytes (d1, NC-059, NC-030; d8, NC-030) and increased expression in activated M1 conditions. Expression results are similar for both monocyte medium and cardiomyocyte medium. CD68 expression was retained in activated M1 throughout d10 (4 days after activation, Scale bar 100µm). response

**
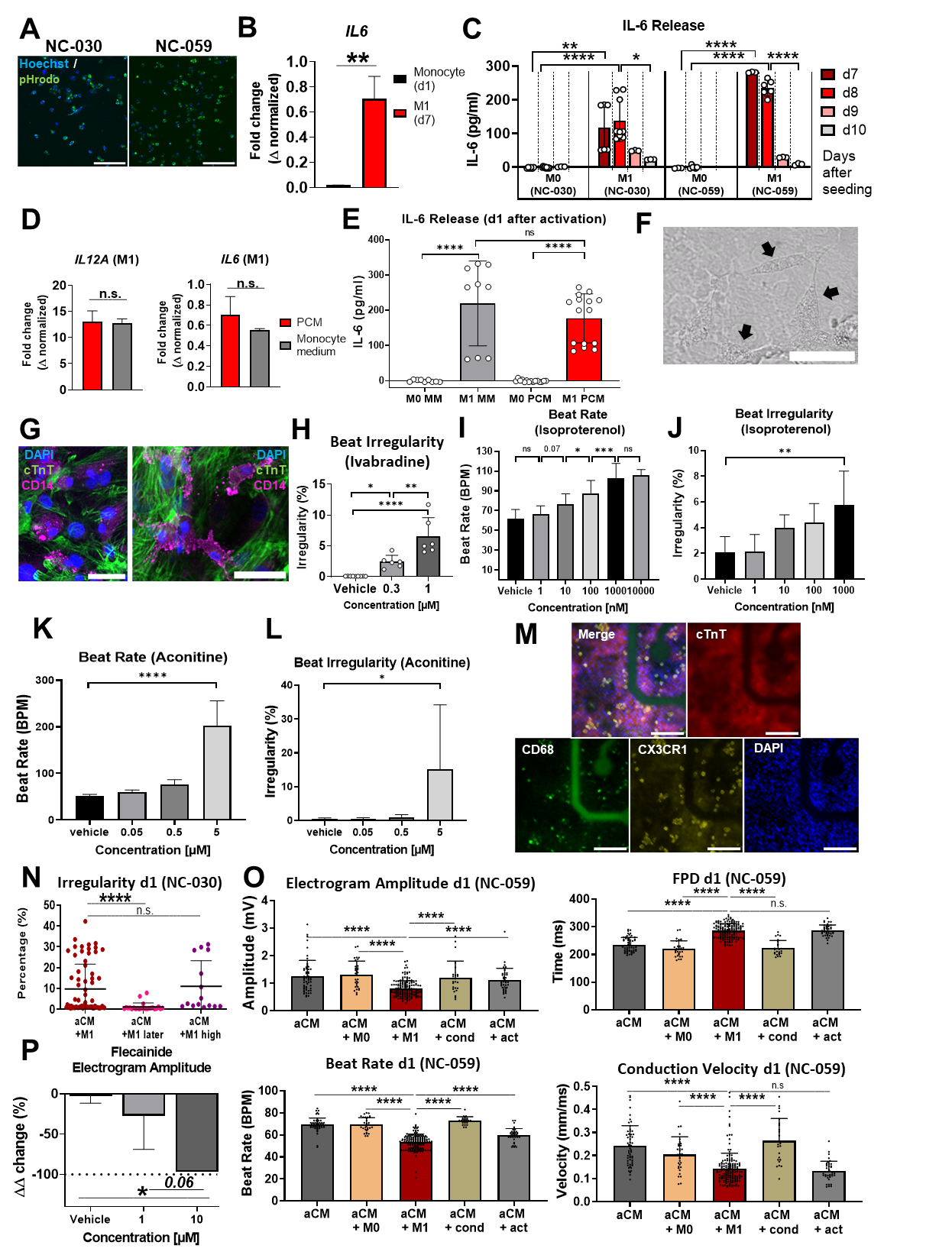
**Figure S4

**Figure S4: Differentiation and activation resulted in pro-inflammatory M1 macrophages in mono- and aCM coculture; aCM+M1 coculture resulted in increased arrhythmia, while its severity was correlated to the timepoint of activation**

**A)** IF images of phagocytic activity showing Hoechst stained M1 macrophages (2 days after activation) taking up pH-activated fluorescent bioparticles into their phagosomes(53). Both lines showed phagocytic activity expressing pHrodo in IF images (scale bar 200µm). **B)** *IL6* expression in RT-qPCR of NC-030 monocytes (d1) and M1 (d7), fold change normalized to GAPDH, showing upregulation in the activated M1 subtype. **C)** IL-6 release in colorimetric assay for NC-030 and NC-059 at different timepoints (d7-d10 after seeding) and subtypes (activated M1 and non-activated M0) cultured in cardiomyocyte medium. Both lines show significantly increased release of IL-6 for M1 compared to M0 and a significant decrease of IL-6 over time after activation at d6 (LoD: <2 pg/mL). **D)** RT-qPCR expression levels for IL12A and IL6 in M1 at 1 day after activation in PCM and monocyte medium. Expression given as fold-change normalized to GAPDH levels. Expression levels did not vary significantly due to different media tested, for either gene. ***E)*** *IL-6 release in colorimetric assay for NC-030 and NC-059 at d1 after activation (M1) and without activation (M0) cultured in either monocyte medium (MM) or cardiomyocyte medium (PCM).* F**)** Brightfield image of NC-030 aCM+M1 coculture, with arrows indicating M1 on aCM monolayer (scale bar 100 µm). **G)** IF images of NC-059 M1 and aCM stained for cTnT, DAPI and CD14, showing CD14^+^ M1 cocultured with aCM (scale bar 50µm). **H)** Compound treatment (aCM, NC-030) using Ivabradine. Pro-arrhythmic Ivabradine showed concentration dependent, significant increase in beat irregularity 48h after addition. One-way Anova used for comparison. ***I)*** *Beat rate after compound treatment (aCM, NC-030) using isoproterenol measured acutely after addition.* ***J)*** *Beat irregularity after acute compound treatment (aCM, NC-030) using isoproterenol.* ***K)*** *Beat rate after compound treatment (aCM, NC-030) using aconitine measured acutely after addition.* ***L)*** *Beat irregularity after acute compound treatment (aCM, NC-030) using aconitine.* **M)** IF images of aCM+M1 coculture on MEA on d10 after seeding (3 days after activation) stained for cTnT, DAPI, CD68 and CX3CR1, showed continued presence of activated macrophages for at least 3 days after activation (NC-030, scale bar 200µm). **N)** Scatter dot plots showing beat irregularity on d1 after activation in aCM+M1 (20,000 + 5,000 cells, respectively) compared to aCM only samples that had activated macrophages added to the well at d0 (also 20,000 + 5,000; aCM+M1 later) and aCM+M1 with higher macrophage cell numbers (20,000 + 10,000) high samples. Addition of activated M1 at d0 did not result in arrhythmia, while the doubling of M1 number did not have an additive effect on the frequency of irregularities. **O)** Bar graphs of NC-059 on d1 after activation comparing conditions for electrogram amplitude, beat rate, FPD and conduction velocity. aCM+M1 showed highly significant changes compared to other conditions, with decreased electrogram amplitudes, lowered beat rates, widened FPDs and reduced conduction velocity. **P)** Compound treatment (aCM, NC-030) using Flecainide. Flecainide showed concentration dependent, significant decrease in electrogram amplitude 0.5h after addition. One-way Anova used for comparison.

Student t-test used for direct comparison, one-way Anova used for time series comparison. All scale bars 100µm if not stated otherwise.

Abbreviations: Cardiomyocyte medium (PCM), atrial cardiomyocytes (aCM), Immunofluorescence (IF), Monocyte medium (MM)

**
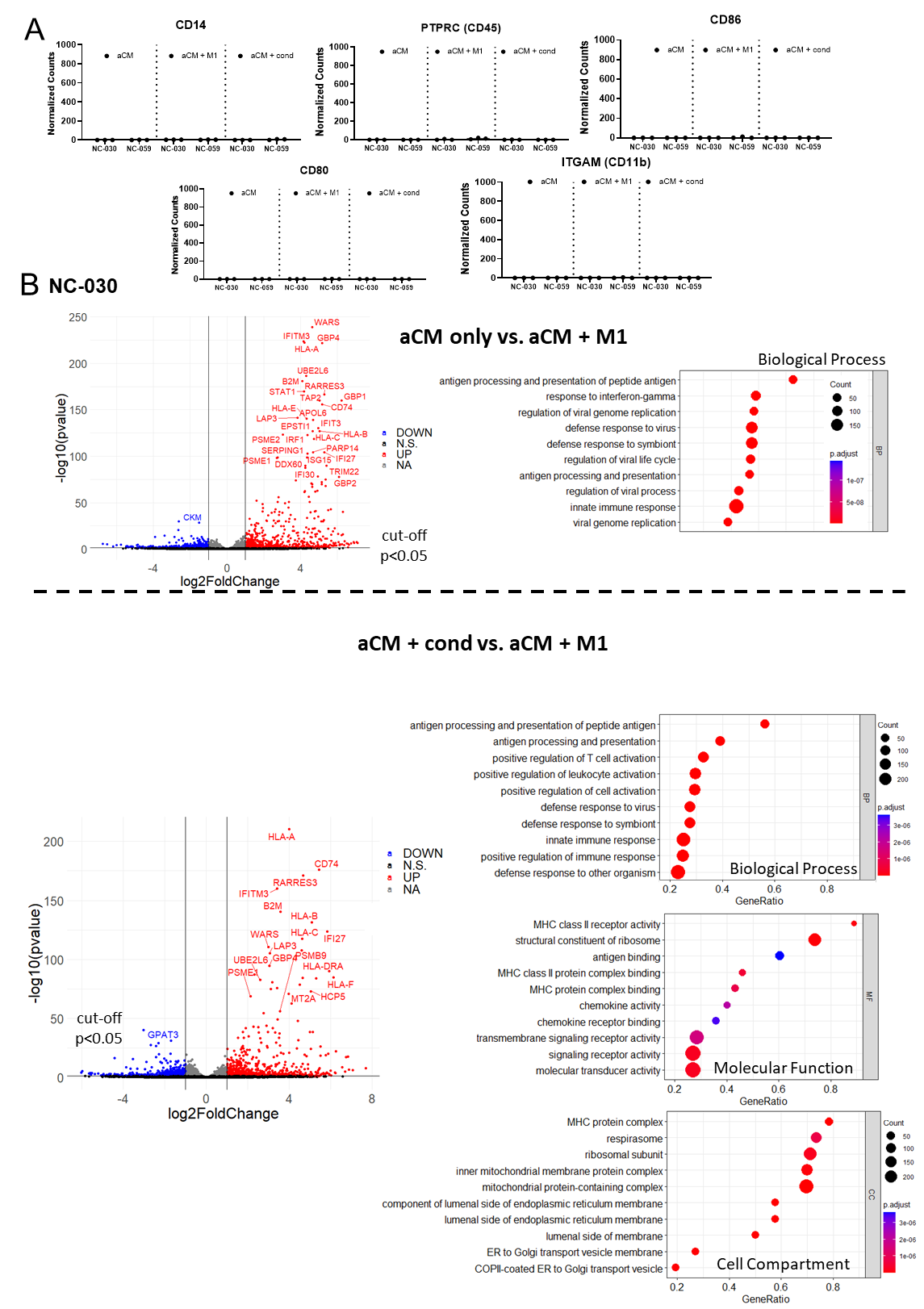
**Figure S5

**Figure S5: Successfully separated cocultures showed increased inflammation-related RNA expression in cocultured aCM compared to control**

**A)** Dot plots with RNA-seq data of NC-059 and NC-030 showing normalized counts of leukocyte/monocyte/macrophage genes in aCM only, aCM+M1 and aCM+cond conditions. Counts for all conditions were minimal, effective removal of macrophages in the aCM+M1 condition using magnetic bead separation of cell populations prior to RNA-seq. **B)** RNA-seq data for NC-030 showing volcano dot plots and gene ontology analysis of aCM only to aCM+M1 (top). Volcano plots show genes significantly (p<0.05) changed as up (fold change >1, red) or down regulated (fold change <-1, blue). Most significantly differentially regulated genes for both lines are upregulated genes compared to aCM only control. Gene ontology analysis highlights inflammation-related biological processes (e.g. response to interferon, antigen processing) as most differentially regulated between conditions. (Bottom) RNA sequencing data for NC-030 showing volcano and dot plots comparing aCM+cond to aCM+M1. Most significantly differentially regulated are upregulated genes compared to aCM+cond. The most differentially regulated processes between conditions show the highly significant upregulation of inflammation-related processes in aCM+M1. aCM+M1 further shows the strong upregulation of MHC related processes in both cell compartment and molecular function.

Abbreviations: atrial cardiomyocytes (aCM), conditioned medium (cond), RNA sequencing (RNA-seq)

**
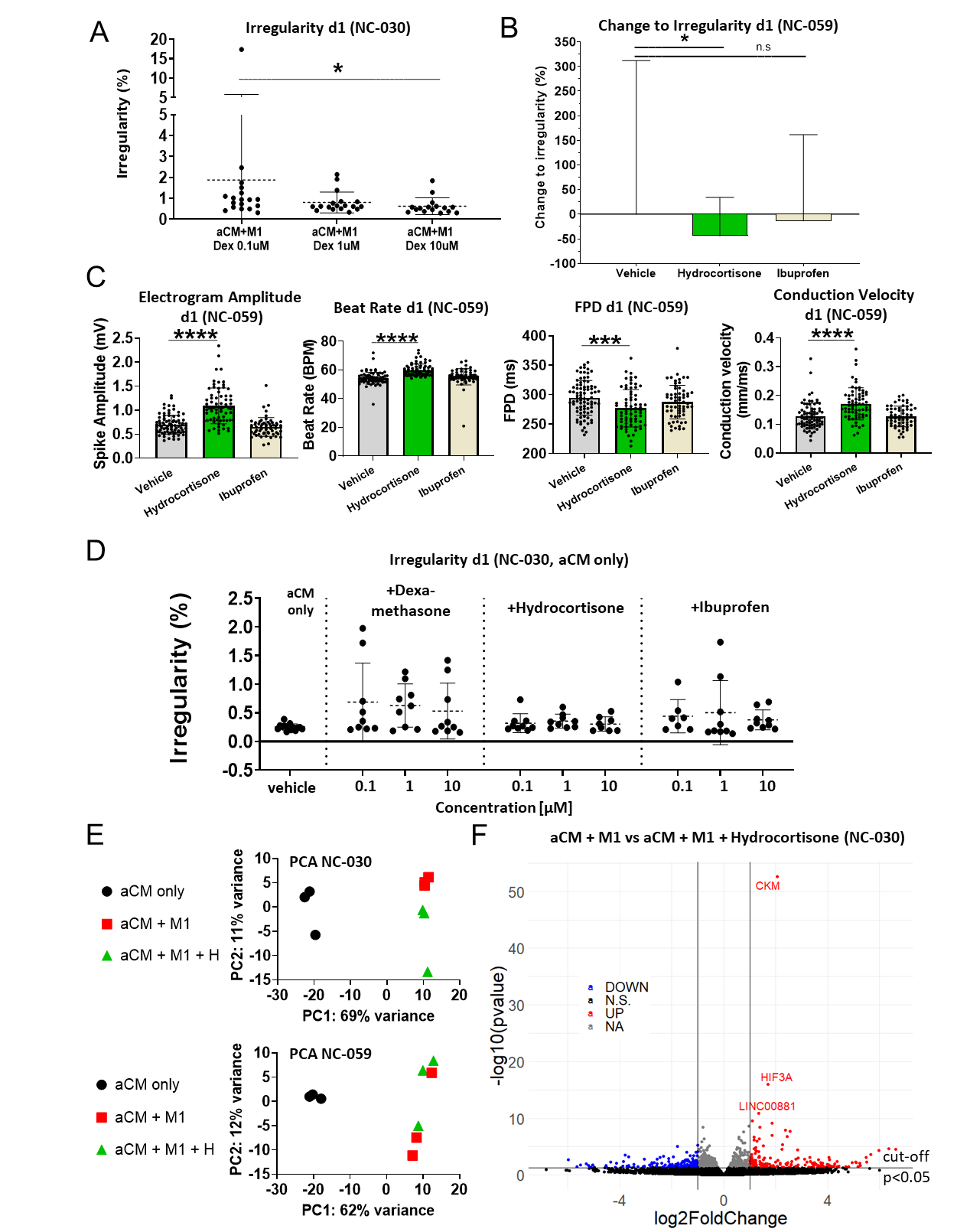
**Figure S6

**Figure S6: Glucocorticoids reduced arrhythmia in cocultures while showing no adverse effects on aCM monocultures**

A**)** Scatter plot showing irregularity for NC-030 aCM+M1 cocultures on d1 after activation and following treatment with differing concentrations of dexamethasone. **B)** Bar graph showing percent change in beat irregularity for NC-059 cocultures on d1 after activation, comparing hydrocortisone and ibuprofen (all 10 µM) to vehicle, with vehicle average taken as baseline. Addition of hydrocortisone resulted in significant irregularity reduction. **C)** Bar graphs showing electrogram amplitude, beat rate, FPD, and conduction velocity for NC-059 for the same conditions. **D)** Dot plot showing irregularity for NC-030 aCM 8 days after seeding and following treatment with multiple concentrations of dexamethasone, hydrocortisone, ibuprofen, or vehicle showing no pro-arrhythmic effect, with all compounds and concentrations showing values far below 5%. **E)** PCA plots of RNA-seq data in NC-030 and NC-059 for the conditions of aCM only, aCM+M1 and aCM+M1+ 10 µM hydrocortisone (aCM+M1+H) separated by the first two principal components. **H)** Volcano plot of RNA-seq data in NC-030 comparing hydrocortisone-treated aCM+M1 to untreated aCM+M1 cocultures.

Abbreviations: atrial cardiomyocytes (aCM), RNA sequencing (RNA-seq), Principal Component Analysis (PCA)

**
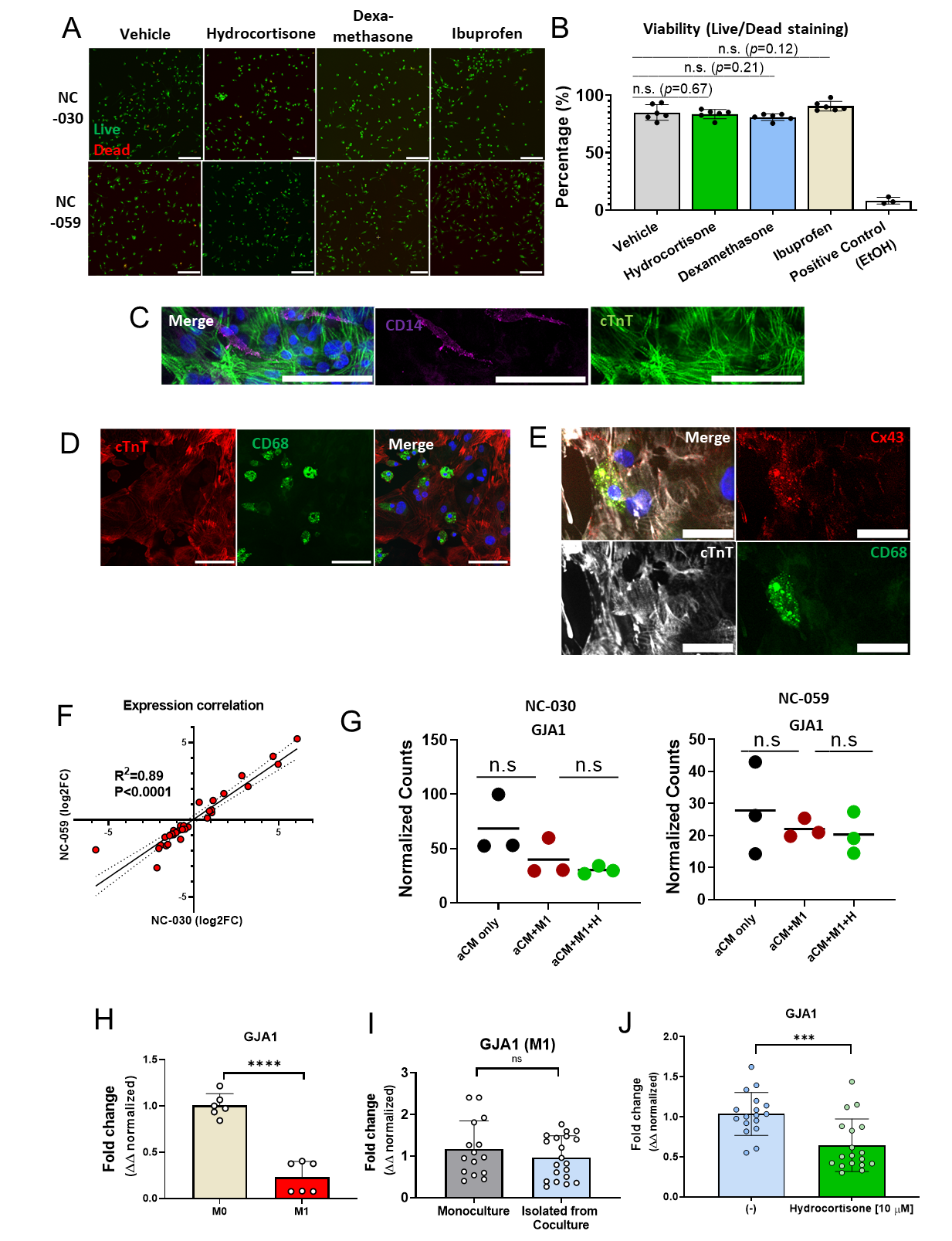
**Figure S7

**Figure S7: Glucocorticoids did not affect M1 viability nor cell identity**

**A)** IF images of NC-030 and NC-059 M1 monocultures 3 days after activation, treated for 4 days with either dexamethasone, hydrocortisone, ibuprofen, or vehicle, and stained with viability dyes, (scale bar 200µm). **B)** Bar graphs showing analysis of IF images of compound treated M1 stained with viability dyes. The amount of living cells is shown in percentage, showing no significant difference between vehicle and the tested compounds. **C)** IF images of NC-059 aCM+M1 coculture 1 day after activation and following treatment with 10 µM hydrocortisone. Cocultures were stained for cTnT, DAPI and CD14 showing retained presence and identity of macrophages after hydrocortisone treatment (scale bar 100µm).  **D)** IF images of NC-059 aCM+M1 coculture 1 day after activation and following treatment with 10 µM hydrocortisone. Cocultures were stained for cTnT, DAPI and CD68 showing retained presence and subtype of macrophages after hydrocortisone treatment (scale bar 100µm). **E)** IF images of NC-059 aCM+M1 coculture 1 day after activation and following treatment with 10 µM hydrocortisone. Cocultures were stained for cTnT, DAPI, Cx43 and CD68, showing retained M1 subtype and Connexin presence (scale bar 20µm). **F)** Expression analysis of identified target genes showing correlation between both donor lines (NC-030 & NC-059) for the aCM only vs aCM+M1 comparison. **G)** Dot plots showing the RNA-seq normalized counts for GJA1 in NC-030 and NC-059. The conditions shown include aCM only, aCM+M1 and aCM+M1+ 10 µM hydrocortisone (+H). ***H)*** *qPCR data of GJA1 expression in M0/M1 monocultures (NC-030, NC-059), normalized to M0 values.* ***I)*** *qPCR data of GJA1 expression in M1 (NC-030, NC-059) in monoculture and in aCM coculture (separated through magnetic beads), normalized to monoculture values.* ***J)*** *qPCR data of GJA1 expression of bead separated M1 cocultured with aCM, untreated (-) and treated with 10 µM hydrocortisone, normalized to untreated.*

Abbreviations: atrial cardiomyocytes (aCM), Immunofluorescence (IF)

Table S1

*Supplementary Table 1: List of genes for NC-030 and NC-059 that are significantly up- or downregulated in aCM+M1 vs aCM only and also significantly, reversely regulated in aCM+M1+Hydrocortisone vs aCM+M1 (Genes shown alphabetically per group)*

| Change in Transcription | Gene Name | Function^^[[1]](#footnote-1)^^ |
| --- | --- | --- |
| Upregulated in aCM+M1 vs aCM only  &  Downregulated in aCM+M1+Hydrocortisone vs aCM+M1 | *A2M* | Inhibitor of proteases and inhibitor/transporter of cytokines |
|  | *CXCL14* | Antimicrobial cytokine involved in immunoregulation and inflammatory processes |
|  | *EPSTI1* | M1 macrophage polarization and regulation of gene expression during M1 versus M2 macrophage differentiation |
|  | *F11R* | Tight junction assembly related; reovirus receptor; leukocyte transmigration; platelet receptor; |
|  | *GBP2* | Interferon-Induced Guanylate-Binding Protein, GTPase that hydrolyzes GTP to GDP |
|  | *GPR37* | G protein-coupled receptor |
|  | *HLA-DPA1* | Immune response related, presents peptides |
|  | *RRAD* | GTP binding activity and calcium channel regulator, suppression of voltage-gated L-type Ca(2+) currents, Inhibits phosphorylation and activation of CAMK2D |
|  | *RSPO3* | Wnt regulation |
|  | *SPON1* | Predicted to be involved with extracellular matrix and cell adhesion |
|  | *TMEM173* | Transmembrane Protein, regulator of innate immune response |
|  | *VCAM1* | Leukocyte cell adhesion |
| Downregulated in aCM+M1 vs aCM only  &  Upregulated in aCM+M1+Hydrocortisone vs aCM+M1 | *ABAT* | 4-aminobutyrate aminotransferase, catabolism of gamma-aminobutyric acid (GABA) |
|  | *ATP1A1* | ATPase, Na+/K+ Transporting, maintaining the electrochemical gradients of Na and K ions across the plasma membrane |
|  | *CPNE5* | Calcium-dependent phospholipid-binding protein, may be involved in calcium-mediated intracellular processes |
|  | *CRYAB* | Chaperone-like activity, preventing aggregation of proteins under stress conditions, dilated cardiomyopathy related |
|  | *FAM13A* | Enabler of GTPase activator activity |
|  | *ITGA7* | Integrin alpha chain, cell-cell / cell-matrix interactions related |
|  | *ITM2A* | Type II membrane protein, suggested to be involved in osteo- and chondrogenic differentiation |
|  | *JHDM1D-AS1* | RNA Gene affiliated with lncRNA |
|  | *KCNA5* | Voltage-gated potassium channel, Kv1.5/I_kur_ |
|  | *KLF9* | Transcription Factor |
|  | *MBTPS1* | Serine protease; cleaves, catalyzes and mediates several proteins including transcription factors |
|  | *METTL7B* | Enables thiol S-methyltransferase activity, predicted involvement in methylation |
|  | *PACSIN1* | Phospholipid binding activity enabler, plasma membrane tubulation |
|  | *PLCD3* | Catalyser of phosphatidylinositol 4,5-bisphosphate hydrolysis, related to increases cytosolic Ca^2+^ concentration |
|  | *SOBP* | Nuclear zinc finger protein, cochlea development related |
|  | *TSPAN7* | Cell surface glycoprotein, related to signal transduction in cell development, activation, growth, and motility |
|  | *VWC2* | May have role in cell adhesion, bone morphogenic protein antagonist |
|  | *ZKSCAN7* | Predicted to enable of DNA-binding transcription factor activity, RNA polymerase II-specific and RNA polymerase II cis-regulatory region sequence-specific DNA binding activity. Predicted to be involved in regulation of transcription by RNA polymerase II |
|  | *ZNF391* | Predicted to enable DNA-binding transcription repressor activity, RNA polymerase II-specific and RNA polymerase II transcription regulatory region sequence-specific DNA binding activity. Predicted to be involved in negative regulation of transcription by RNA polymerase II. |

Table S2

*Supplementary Table 2: List of Antibodies (ordered by mention)*

| Name | Target | Dilution | Supplier | Species | Reactivity |
| --- | --- | --- | --- | --- | --- |
| cTnT Reafinity conjugated FITC | cTnT | 1:10 (Flow cytometry) 1:50, 1:100 (IF) | Miltenyi | Human | Human |
| MLC2a Reafinity conjugated APC | MLC2a | 1:10 (Flow cytometry) | Miltenyi | Human | Human |
| REA control FITC | - | 1:10 (Flow cytometry) 1:50, 1:100 (IF) | Miltenyi | Human | - |
| REA control APC | - | 1:10 (Flow cytometry) 1:50 (IF) | Miltenyi | Human | - |
| Human COUP-TF II/NR2F2 Antibody | COUP-TF II | 1:100 | R&D Systems | Mouse | Human |
| (APC) AffiniPure F(ab')₂ Fragment Donkey Anti-Mouse IgG (H+L) | - | 1:500 | Jackson ImmunoResearch | Donkey | Mouse |
| Purified Mouse IgG2a, κ | - | 1:100 | BioLegend | Mouse | - |
| CD45 PE | CD45 | 1:20 | BioLegend | Mouse | Human |
| CD11b APC | CD11b | 1:20 (Flow cytometry) 1:50 (IF) | BioLegend | Mouse | Human |
| CD14 FITC | CD14 | 1:20 (Flow cytometry) 1:100 (IF) | BioLegend | Mouse | Human |
| IgG1-PE | - | 1:160 | BioLegend | Mouse | - |
| IgG1-APC | - | 1:40 (Flow cytometry) 1:100 (IF) | BioLegend | Mouse | - |
| IgG1-FITC | - | 1:10 (Flow cytometry) 1:50 (IF) | BioLegend | Mouse | - |
| Vimentin REAfinity™ conjugated FITC | Vimentin | 1:50 | Miltenyi | Human | Human |
| CD68 (eBioY1/82A (Y1/82A)) conjugated FITC | CD68 | 1:200 | Invitrogen | Mouse | Human |
| CX3CR1 (1H14L7) | CX3CR1 | 1:250 | Invitrogen | Rabbit | Human |
| IgG2b-FITC | - | 1:1600 | Invitrogen | Mouse | - |
| IgG | - | 1:1500 | Invitrogen | Rabbit | - |
| IgG (H+L) Alexa Fluor 488 | - | 1:500 | ThermoFisher Scientific | Goat | Human |
| IgG (H+L) Alexa Fluor 594 | - | 1:200 | ThermoFisher Scientific | Donkey | Rabbit |
| IgG (H+L) Alexa Fluor 488 | - | 1:200 | ThermoFisher Scientific | Donkey | Mouse |
| IgG2b Alexa Fluor 647 | - | 1:200 | ThermoFisher Scientific | Goat | Mouse |
| cTnT REAfinity conjugated APC | cTnT | 1:50 | Miltenyi | Human | Human |
| Connexin 43 ZooMAb® | Connexin 43 | 1:50 | Sigma-Aldrich | Rabbit | Human |
| IgG1 Alexa Fluor 555 | - | 1:500 | ThermoFisher Scientific | Goat | Mouse |
| MLC2a (Monoclonal Mouse Antibody [56F5] | MLC2a | 1:500 | Synaptic Systems | Mouse | Human |
| MLC2v Pab | MLC2v | 1:150 | PreoteinTech Group | Rabbit | Human |
| IgG Alexa Fluor 647 | - | 1:500 | ThermoFisher Scientific | Chicken | Rabbit |

Video S1

Brightfield video of NC-030 aCM+M1 coculture, with arrows indicating M1 on aCM monolayer (scale bar 100µm)

1. Adapted from GeneCards®: The Human Gene Database, https://www.genecards.org/ (22. Mar. 2023) [↑](#footnote-ref-1)
